# Transcriptional reprogramming of the stress response and translational-secretory machinery enhances recombinant secreted protein production in *Yarrowia lipolytica*

**DOI:** 10.1186/s12934-026-03062-2

**Published:** 2026-07-20

**Authors:** Maria Gorczyca, Piotr Kubiak, Ewelina Celińska

**Affiliations:** Department of Biotechnology and Food Microbiology, Poznan University of Life Sciences, Wojska Polskiego 48, Poznan, 60-637 Poland

**Keywords:** Global transcription reprogramming, Transcription factor, Recombinant protein synthesis, Ribosomal-translational capacity, Yeast

## Abstract

Protein synthesis and secretion processes are non-pathway traits. The genetic complexity makes them very challenging to engineer and genetically optimize. Therefore, we exploit transcriptional reprogramming to create a favorable cellular environment for a trait of interest. In this study, we investigate the effects of transcription factor (TF) engineering on secretory recombinant protein (rProt) production in *Y. lipolytica*. High-throughput genome-wide pre-screening guided the TFs selection. Here, we run high-throughput functional studies, followed by transcriptome analysis for selected TF-modified strains from semi-steady state. We contrasted the overexpression (OE) vs. deletion (KO) genotypes for their capacity to produce two reporter proteins (fluorescent model and glucoamylase). Despite a wide set of TF-modified strains analyzed here, only several displayed consistent, unbiased phenotypes. Collected data indicated that the known general stress response TFs, Msn4 and Hsf1, elicit their highly beneficial rProt-promoting function specifically towards a “challenging” rProt, while the levels of “simple” rProt remain unaffected. Deletion of the previously identified strong repressor of intracellular rProt synthesis, Azf1, significantly enhanced production of secretory rProt. At the transcriptomic level, deletion of Azf1 was associated with massive upregulation of ribosome biogenesis and cytoplasmic translation, which underwent alternative splicing (global differential exon usage analysis). The results of contrasting the Azf1- and Dep1-driven regulomes (the latter identified as a strong repressor of intracellular rProt synthesis, with no positive effect on the secretory rProt production) suggest that for efficient rProt secretion, upregulation in the vesicle-mediated transportation must co-occur, as in the case of Azf1-KO. In our experimental setting, OE of the general stress response TFs was beneficial for the production of “problematic”, UPR-awakening rProt, and deletion of Azf1 for tuning a molecular background beneficial for secretory rProt.

**Graphical Abstract:**

**Figure:**
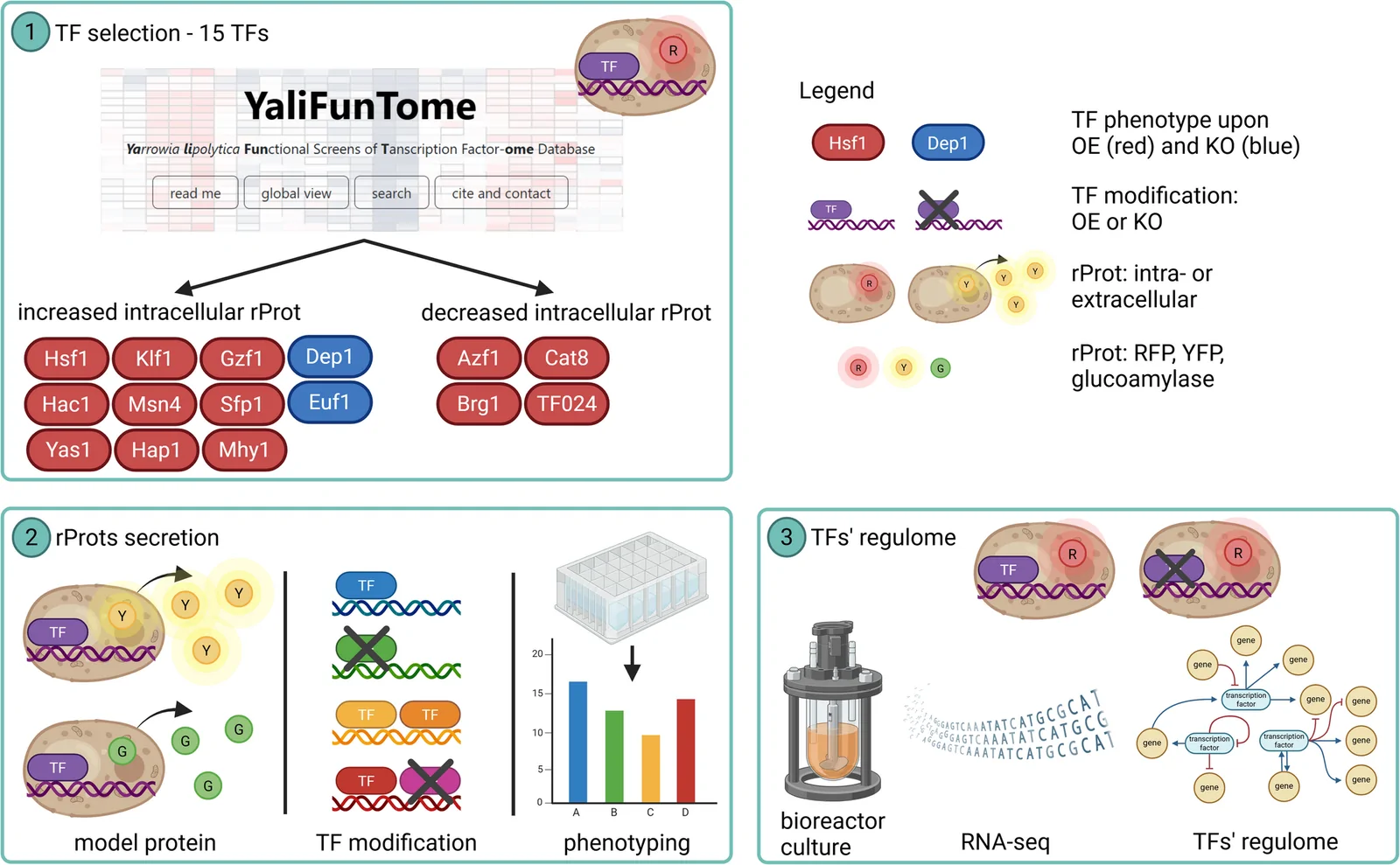

**Supplementary Information:**

The online version contains supplementary material available at https://doi.org/10.1186/s12934-026-03062-2.

## Background

Protein is a unique product of biotech processes. Unlike fuel or small-molecular-weight chemicals, it cannot be outsourced from any other source than a living organism. Proteins are produced by living organisms, and they elicit their most precious functions within them. At present, chemical synthesis cannot compete with biology in supplying biologically active proteins sustainably, which forms the rationale behind our studies on recombinant proteins (rProts) production in microbial hosts.

In our research, we use *Yarrowia lipolytica* yeast as the rProt production platform. We are mainly interested in secretory proteins, for obvious convenience in downstream processing and higher yields [1–3] . In general, polypeptides tagged for secretion are processed via different machinery and traverse different compartments when compared to their cytoplasmic counterparts. Multiple discrete molecular characteristics make *Y. lipolytica* an “advanced secretor”, such as preference for co-translational translocation rather than the less efficient post-translational translocation, operable calnexin cycle, specific elements typical for higher eukaryotes (for details, please go to [4]). Direct comparisons with the other yeast species showed that *Y. lipolytica* is an attractive platform for secretory rProt production [5, 6].

The protein synthesis and secretion processes are non-pathway traits. The genetic complexity makes them very challenging to engineer and genetically optimize. Literature reports demonstrated the usefulness of global transcription machinery engineering (gTME, or global transcriptional reprogramming [7]) for enhancing rProt synthesis in *Saccharomyces cerevisiae* [8] or *Komagataella phaffi* [9, 10]. Recently, we have used gTME to identify facilitators and repressors of rProt biosynthesis in *Y. lipolytica* [11–14] (YaliFunTome database: https://sparrow.up.poznan.pl/tsdatabase/). These were transcription factors (TFs) or chromatin remodellers that enhanced or limited yields of an intracellular reporter rProt. It remains to be settled whether they will maintain their beneficial effects when secretory rProt will be used as the reporter, which is the main aim of this study.

**Fig. 1:**
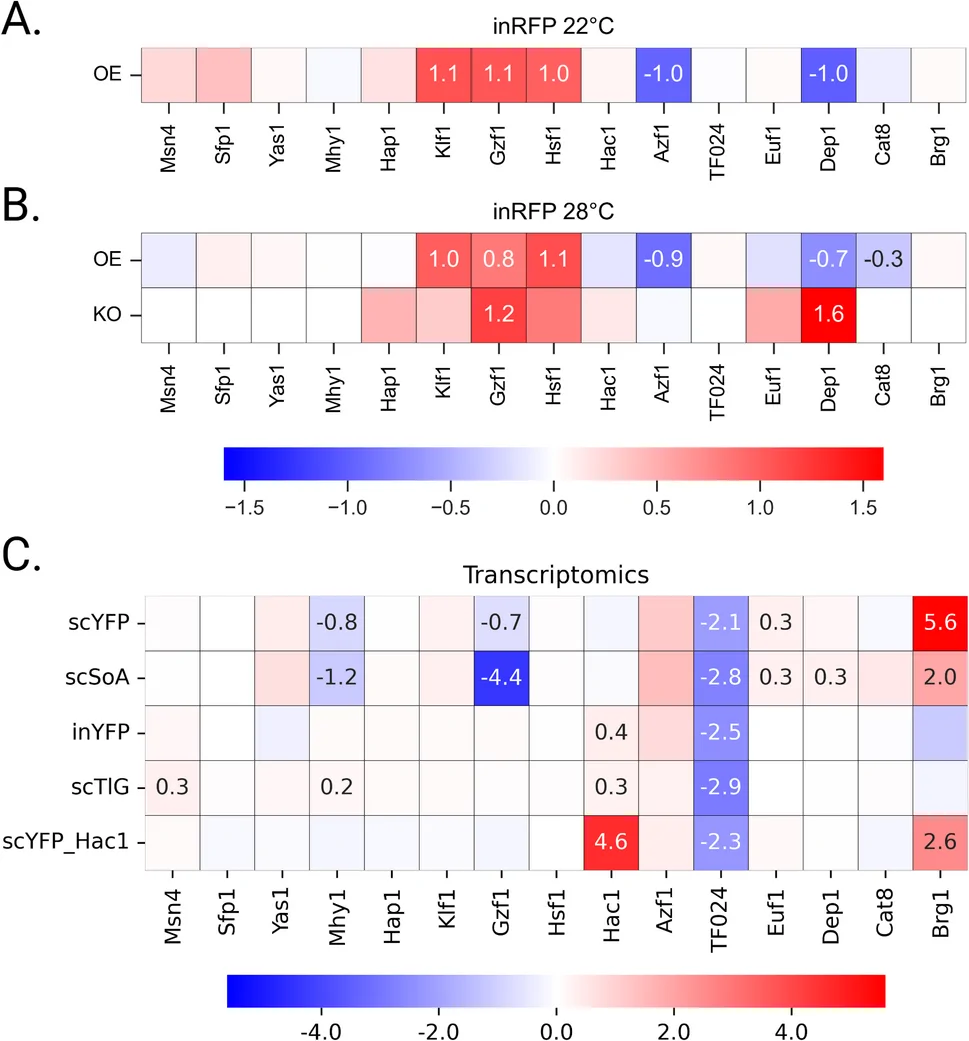
Summary of the previous results on the fifteen TFs’ performance in a form of heatmap. **A** impact of selected TFs’ co-overexpression on intracellular accumulation of RFP, analyzed under 22 °C. **B** impact of selected TFs’ co-overexpression (OE) or deletion (KO) on intracellular accumulation of RFP, analyzed under 28 °C. **C** level of a given TF’s expression in *Y. lipolytica* cells overexpressing different rProts. Phenotyping data were withdrawn from YaliFunTome database (https://sparrow.up.poznan.pl/tsdatabase/). Transcriptomics data were recycled from SRA deposited data. Numbers in the heatmaps cells represent the RC values for nsFL results (**A**, **B**) or RC in gene expression levels (**C**) over the control strain. Whenever the RC was statistically significant – the numerical value is displayed. Heatmaps are color-coded according to provided legends

The selection of the TFs to be studied here was based on the previous results of high-throughput overexpression screens [12], and their transcriptional profiles were studied via a data-recycling approach [14]. Table 1 lists the TFs with their known/putative function, and so far established effect on the rProt synthesis or stress resistance. A summary of the TFs’ performance in the previous experiments is compiled in Fig. 1.

In this research, we investigate the effects of fifteen TFs on secretory rProt production in *Y. lipolytica* by running conventional, functional studies: overexpression (OE) and knock-out (KO) of the gene of interest. Here, we adopted high-throughput but reliable phenotyping [15] to gain deeper insight into the TFs’ operation. We moved from intracellular rProts used previously to rProts targeted for secretion. Additionally, apart from using only a convenient fluorescent reporter (scYFP), we included a rProt of industrial relevance (glucoamylase; scTlG), known to induce high cellular stress, and synthesized in limited amounts in *Y. lipolytica* [1, 16, 17].

**Table 1:** List of TF genes studied. Custom name (if available) and reference to the Yali0 and Yali1 versions of the genome are given. Links direct to specific pages in the YaliFunTome database showing previous results on the TF’s phenotyping

Gene	Yali0 number	Yali1 number		YaliFunTome results (OE)
Msn4	YALI0C13750g	YALI1_C19151g	Msn4 belongs to the Msn2/4 zinc finger family, binding to cis-acting DNA stress response elements (STRE). In *Y. lipolytica*, two Msn2/4 family representatives are known: Mhy1 and Msn4. Previously, neither OE nor KO of Msn4 led to an apparent phenotype in *Y. lipolytica* for intracellular rProt production. Yet, OE of Msn4 (and its synthetic version synMsn4, alone or in combination) enhanced rProt production in *K. phaffi* [18].	https://sparrow.up.poznan.pl/tsdatabase/?page=gene&name=TF107
Mhy1/Msn2	YALI0B21582g	YALI1_B28150g	Msn2/Msn4-like protein, a key regulator of yeast-to hypha dimorphic transition but not stress resistance, regulates both alkaline-pH and glucose-induced filamentation. Regulated by the kinase Rim15p, which itself is repressed by the Tor nitrogen signaling pathway. Regulome described [19–22]. Previously, neither OE nor KO of Mhy1 led to an apparent phenotype in *Y. lipolytica* for intracellular rProt production.	https://sparrow.up.poznan.pl/tsdatabase/?page=gene&name=TF095
Sfp1	YALI0F11487g	YALI1_F15218g	No similarity-based annotation available	https://sparrow.up.poznan.pl/tsdatabase/?page=gene&name=TF123
Yas1	YALI0C02387g	YALI1_C03349g	Transcription factor essential for cytochrome p450 induction in response to alkanes. A component of the heteromeric Yas1p/Yas2p complex transcription factor. Previously found to endow *Y. lipolytica* with low pH and high temperature stress resistance [13].	https://sparrow.up.poznan.pl/tsdatabase/?page=gene&name=TF083
Hap1	YALI0F17424g	YALI1_F23191g	Hap1 is implicated in regulating aerobic metabolism in S. cerevisiae. It is involved in sensing O2 levels via the heme signaling pathway, and in response activates the oxidative stress response-associated genes [23]. The OE of HAP1 in S. cerevisiae attenuated the negative impacts of oxidative stress and elevated the production of the target rProt [24].	https://sparrow.up.poznan.pl/tsdatabase/?page=gene&name=TF120
Klf1	YALI0D05041g	YALI1_D06489g	Krueppel-like factor 15, regulates the expression of genes for gluconeogenic and amino acid-degrading enzymes. Klf1 was extensively studied in *Schizosaccharomyces pombe* during long-term G0 quiescence induced by N starvation [25]. Expression of KLF1 was induced under N starvation, while ∆klf1 mutants exhibited changes in cell morphology and were unable to restore cell division. It specifically reacts to oxidative stress via nuclear relocalization [26]. The regulome of the schpKlf1 covers genes implicated in cell wall renewal, oxidative stress response, glycolysis, nutrient uptake, RNA-mediated chromatin silencing, glycosidation, and methylation [25]. Previously identified as a universal enhancer of intracellular rProt synthesis [12].	https://sparrow.up.poznan.pl/tsdatabase/?page=gene&name=TF126
Gzf1	YALI0D20482g	YALI1_D26006g	Inducer of the nitrogen catabolite repression (NCR) genes. The repressor Gzf3 and the activator Gzf2 were shown to regulate GZF1’s expression, suggesting that, even though no nitrogen-dependent phenotype is exposed by Gzf1-KO, Gzf1 plays a role in nitrogen source-specific gene regulation that may be partially redundant with Gzf2 [27]. Gzf1 was found to be a universal (condition-independent) inducer of rProt synthesis, which did not require any specific external inducer to elicit its beneficial action [12].	https://sparrow.up.poznan.pl/tsdatabase/?page=gene&name=TF037
Hsf1	YALI0E13948g	YALI1_E16974g YALI1_E16982g	Hsf1, was previously found to be a potent, universal enhancer of intracellular rProt synthesis [12]. It is one of the most frequently used gTME tools for successful enhancement of rProt synthesis [28–30].	https://sparrow.up.poznan.pl/tsdatabase/?page=gene&name=TF068
Hac1	YALI0B12716g	YALI1_B16808g	Transcriptional activator of genes involved in ER-based Unfolded Protein Response (UPR). Regulome described in [31].	https://sparrow.up.poznan.pl/tsdatabase/?page=gene&name=TF006
Azf1	YALI0A16841g	YALI1_A16891g	In *S. cerevisiae* and *Ogataea polymorpha*, Azf1 acts as a carbohydrate-sensing TF, which activates genes involved in growth, carbon metabolism, and cell wall organization in response to glucose [32, 33]. In *Y. lipolytica*, OE of Azf1 led to 30% decrease in storage lipid concentration [34, 35]; which consistently supports its role in carbon catabolism and respiratory metabolism. Previously, the Azf1-KO genotype rendered no significant phenotype in terms of intracellular rProt levels, while its OE triggered a reduction in this parameter [12, 14].	https://sparrow.up.poznan.pl/tsdatabase/?page=gene&name=TF124
TF024	YALI0D01353g	YALI1_D01548g	No similarity-based annotation available	https://sparrow.up.poznan.pl/tsdatabase/?page=gene&name=TF024
Euf1	YALI0F01562g	YALI1_F02434g	Transcription factor mediating expression of erythritol synthesis genes. Regulome described [36]. Used in a synthetic circuit for erythritol-inducible rProts synthesis [37].	https://sparrow.up.poznan.pl/tsdatabase/?page=gene&name=TF054
Dep1	YALI0F05896g	YALI1_F08745g	Dep1 (Deregulated Expression of Phospholipid biosynthesis) was previously found to enhance the accumulation of lipids in *Y. lipolytica* [34, 35]. Regarding its role in the other species, Dep1 was shown to activate phospholipid biosynthesis in Fusarium sp. [38], but inhibit effectors of phospholipid synthesis in *S. cerevisiae* [39]. It was thus deemed a transcriptional modulator involved in the regulation of structural phospholipid biosynthesis genes. Structurally, Dep1 is a part of the Rpd3C(L) histone deacetylase complex (HDAC). Considering all these results and Dep1’s implication in both rProt synthesis and lipid accumulation (a trait of great interest), we decided to analyze Dep1’s transcriptome.	https://sparrow.up.poznan.pl/tsdatabase/?page=gene&name=TF128
Cat8	YALI0C19151g	YALI1_C26501g	CATabolite repression TF 8. Inducer of gluconeogenesis, maintains energy homeostasis, presumed to regulate formate dehydrogenases expression [40]. iModulon available [41, 42].	https://sparrow.up.poznan.pl/tsdatabase/?page=gene&name=TF122
Brg1	YALI0E31757g	YALI1_E37574g	Biofilm regulator 1. Was found to induce lipid synthesis/accumulation in *Y. lipolytica* [34, 35].	https://sparrow.up.poznan.pl/tsdatabase/?page=gene&name=TF053

## Methods

### Microbial strains and routine maintenance

All the *Y. lipolytica* strains used in this study are derivatives of either: (i) Po1f (*MatA*,* leu2-270*,* ura3-302*,* xpr2-322*,* axp-2*; phenotype: ΔAEP, ΔAXP, ura-, leu-; ATCC MYA-2613) or (ii) JMY8695 (*MatA ura3-302*,* leu2-270*,* xpr2-322*,* Δlip2*,* Δlip7*,* Δlip8*,* Δlys5*,* Δeyk1*,* Δmhy1*; Ura−, Leu-, Lys−, Fil-, Ery utilization-) [43]. These could be modified in TF *loci* or the reporter secretory proteins (secretory YFP, scYFP; or secretory glucoamylase from *Thermomyces lanuginosus*, scTlG). Six control strains were used as references to assess the change driven by TF-modification: (1) and (2) positive control (“control +“): Po1f derivative overexpressing (1) scYFP or (2) scTlG in JMP62-URA3ex vector, remaining auxotrophy complemented with an empty vector JMP62-LEU2ex vector, (3) negative control (“control –“): Po1f-derivative with both auxotrophies complemented with empty vectors; (4) and (5) double positive control (“control double +“): JMY8695 derivatives overexpressing 4. scYFP or 5. scTlG in JMP62-URA3ex vector, remaining auxotrophy complemented with an empty vector JMP62-LEU2ex vector and Lys5 solo cassette, (6) double negative control (“control double -“): JMY8695 derivatives with three auxotrophies complemented with empty vectors. Nr 1, 2, and 3 - control strains relevant for strains with a single TF modified, numbers 4, 5, and 6 - control strains relevant for strains with two TFs modified. *Escherichia coli* was used for vector construction and subcloning. The full list of all the strains is given in Tables S1 and S2. The list of the final *Y. lipolytica* strains phenotyped in this work is provided in Table S3.

*Y. lipolytica* and *E. coli* strains were maintained according to standard protocols [44, 45]. *E. coli* DH5α and its derivatives were grown in LB medium (g/L: yeast extract, 5 (Biomaxima, Poland), bacteriological peptone, 10 (Biocorp, Poland), NaCl, 5 (POCh, Gliwice, Poland)), supplemented with kanamycin (40 µg/mL) or ampicillin (100 µg/mL), and agar, 15 (BTL, Poland), when required. Yeast strains were routinely maintained in YNB medium (g/L: YNB w/o amino acids (AA) and ammonium sulfate (AS) 1.7 (Merck-Millipore), ammonium sulfate 5 (PoCh), glucose 20 (PoCh)), or in YPD medium (g/L: yeast extract 10, bacteriological peptone, 20, glucose, 20), solidified with agar, 15, when required. Yeast liquid cultures were run in 24-square-well plates and shaken at 250 rpm, 28 °C. Bacterial liquid cultures were run in 15 mL glass tubes and shaken at 200 rpm, 37 °C.

### Molecular biology techniques and reagents

*E. coli* and *Y. lipolytica* transformations followed standard protocols, heat-shock and lithium acetate heat-shock, respectively, as described in [44, 45]. Restriction endonucleases (*AvrII*, *BamHI*, *NotI*, *EcoRI*, *KpnI;* Thermo Fisher Scientific, Waltham, Massachusetts, USA), Phire DNA polymerase (Thermo Fisher Scientific), T4 DNA ligase (New England Biolabs, Ipswich, USA), and pCR Blunt II TOPO vector (Thermo Fisher Scientific) were all used according to protocols provided by the respective manufacturers. DNA plasmid isolation, DNA fragment extraction from agarose gel, and purification were performed with corresponding kits from A&A Biotechnology (Gdynia, Poland).

The TFs studied here are listed in Table 1. Full DNA sequences of the TFs to be OE (codon-optimized, synthetic versions) are listed in Table S4. All the oligonucleotides used for amplification of the TFs, or homologous arms from genomic DNA, are listed in Table S5.

### Cloning strategy

The background strains were constructed by transforming the Po1f or JMY8695 strains with an empty JMP62-URA3ex cassette or the JMP62-URA3ex vector, bearing a specific secretory reporter protein – either a secretory version of Yellow Fluorescent Protein (scYFP) or secretory glucoamylase from *T. lanuginosus* (scTlG). These were then either transformed with the LEU2 cassette (JMP62-LEU2ex) (and additionally with JMP62-LYS5ex for JMY8695) to generate a background strain for deletions (KO strains) or as a control strain. Each time, bacterial parts of the shuttle plasmids were removed before transformation by digestion with the *NotI* restriction enzyme (JMP62-derivatives; OE) or *EcoRI* and *NotI* (pV2-derivatives; KO).

#### TF overexpression

The TF genes were either directly amplified from genomic DNA or purchased from GeneCust (Boynes, France) as synthetic DNA fragments in the pUC57 vector (kindly donated by Prof. Jean-Marc Nicaud from INRAE, Jouy-en-Josas, France). Genes *MSN4* and *YAS1* were amplified from genomic DNA with specific flanks *BamHI* at 5’ and *AvrII* at 3’ ends added (Table S5). *MHY1*, *HSF1*,* KLF1*,* GZF1*,* SFP1*,* HAC1*, and *HAP1* were purchased as synthetic DNA fragments, due to internal endonuclease recognition sites, further codon-optimized, and with the respective overhangs at the termini. Of note, *HSF1* (mutation R68S introduced), *KLF1* (removal of two introns – 84 and 497 bp - identified previously [46], *SFP1* (803 bp intron removed), and *HAC1* (removal of 30 bp intron identified previously [31, 47, 48]) were additionally modified based on the data from previous studies (full sequences used in this study are given in Table S4; schematic representation of introns and an activation site on Supplementary Fig. S1). After subcloning into the pCR Blunt II TOPO vector, the TF-encoding fragments were cloned into JMP62-LEU2ex under the control of the constitutive pTEF promoter.

The transformations were run in consecutive rounds to increase the effectiveness and number of correct clones. Firstly, the parental strain (Po1f or JMY8695) was transformed with the JMP62-URA3ex vector, bearing a specific secretory reporter protein – either scYFP or scTlG, followed by plating on YNB Drop-Out medium (Drop-Out without uracil; Y1501, Merck). The second round of transformation was run with JMP62-LEU2ex-TF and plated onto YNB Drop-Out medium (Drop-Out without histidine, leucine, tryptophan, and uracil; Y2001, Merck). At least 12 clones (up to 24) were picked after 2 days of incubation at 28 °C for screening.

#### TF deletion

The KO cassettes were constructed on the backbone of the pV2 vector [49]. The scheme is shown in Fig. S2. First, the nourseothricin resistance gene (NATr) was cloned between the *KpnI* and *BamHI* recognition sites to generate the pV2-NATr vector. The homologous arms flanking the NATr gene were amplified from the Po1f strain genomic DNA for *TF024*,* BRG1*,* EUF1*,* CAT8*,* DEP1*, and *AZF1*. Around 1000 bp were amplified upstream/downstream ATG/stop codon, respectively (taking care not to interfere with adjacent ORFs). Upstream homologous arm (ArmUP) was flanked with *EcoRI* and *KpnI*, and the downstream arm (ArmDown) with *BamHI* and *NotI*, recognition sites at 5’ and 3’ ends, respectively. The individual fragments for homologous arms were first subcloned into the pCR Blunt II TOPO vector and further cloned into pV2-NATr. The correct assembly of expression cassettes was verified by PCR, enzyme digestion, and sequenced (Genomed, Warsaw, Poland). The ArmUP-NATr-ArmDown cassette was released from pV2-NATr by digestion with *EcoRI* and *NotI* endonucleases and transformed into one of the *Y. lipolytica* parental strains. The transformants were plated onto YPD NAT (100 µg/ml) and incubated at 28 °C. At least 5 clones were picked after 2–3 days of incubation at 28 °C for screening. All the positively verified strains were deposited as 15% glycerol stocks at -80 °C.

### Culturing and downstream analysis

#### Growth conditions

After the initial screening of 5–24 clones, three representative clones with the most frequent growth phenotype were subjected to the main cultures. Due to high nutrient requirements caused by the production of the secretory reporters (scYFP, scTlG), the main cultures were run in rich YPG20/YPG100 medium (g/L: yeast extract, 10, bacteriological peptone, 20, glycerol, 20/100), buffered at pH 5.0 with maleic buffer for YPG20. The cultures for scTlG production were not buffered with maleic buffer, as it was shown to inhibit scTlG activity (not shown). From our previous studies, we know that simple YNB does not sufficiently support secretory protein production by *Y. lipolytica*, and as demonstrated recently, it undergoes batch-to-batch variability [50]. The batch cultivations were conducted in the 24-well Deutz system plates, shaken at 250 rpm, at 22 °C and 28 °C [15]. The temperature was selected based on previous results showing that decreased temperature (from 30 °C to < 25 °C) significantly enhances secretory protein production per cell [51]. The adopted temperature did not negatively impact the strains’ growth. Samples were collected at 48 h of cultivation, being the most representative time point [15]. The representative clones for each genotype were cultured in at least biological duplicates and measured in technical duplicates.

#### Sample processing and analytical methods

##### Growth

Biomass samples were diluted in 0.75% NaCl to fit into the linear range of the absorbance measurement (absorbance at 600 nm wavelength; < 1.0). Measurements were run in a 96-well flat-bottom transparent MTP plate at 600 nm in the Tecan Spark plate reader (Tecan Group Ltd., Mannedorf, Switzerland).

##### Fluorescence

All the fluorescence measurements were conducted in black flat-bottomed MTP plates (Corning; Sigma Aldrich, Merck) in the Tecan Spark plate reader. The wavelength settings (excitation/emission): scYFP 510/590 nm. The extracellular YFP fluorescence was measured in the supernatant samples. Fresh culture medium was used as the background reference. The intracellular YFP fluorescence was measured in the pre-washed biomass samples, resuspended and diluted in sterile saline solution.

##### Enzymatic assay microDNS

The glucoamylase activity was determined in supernatant samples according to the previously developed [52] and subsequently customized microDNS assay [53]. One activity unit (glucoamylase activity unit; [GAU]) was defined as the amount of enzyme that releases 1 µg of reducing sugar equivalents per 1 mL, during 1 h, at pH 5.0 and 40 °C, under the adopted assay conditions, read at 540 nm. The supernatant from the reference strain cultures was assayed under the same conditions to assess any possible background activity. All the readouts were normalized per blank reaction with medium. Each sample was analyzed in technical duplicates.

### Data processing and statistical analysis

Representative subclones for each genotype were selected from the subclones screening cultures using a custom Python script. With the use of a clustering algorithm, the clones with the predominant phenotype were selected.

The fluorescence or activity results were normalized per biomass amounts determined via OD600, rendering “specific fluorescence”/”specific activity” metrics (sFL [FL_analyzed_/OD_analyzed_], sGAU [GAU_analyzed_/OD_analyzed_]). The reference strains (no reporter proteins) were used for the background normalization (rendering normalized metrics – nsFL [sFL_analyzed_-sFL_reference_] or nGAU [sGAU_analyzed_-sGAU_reference_]). Relative change (RC) in FL or GAU of the studied TF was calculated in relation to the positive control strain [(nsFL_analyzed_-nsFL_control_)/nsFL_control_] or [(nsGAU_analyzed_-nsGAU_control_)/nsGAU_control_]. Data normality was assessed using the Shapiro–Wilk test. As at least one group violated the normality assumption, non-parametric methods were applied. Group differences were evaluated using the Kruskal-Wallis test followed by Dunn’s post-hoc comparisons, with Benjamini-Hochberg correction for multiple testing. Results are reported as median and interquartile range (IQR). All the analyses were performed in Python 3.11.9, with visualizations in Python or R 4.1.2.

### Transcriptomics

#### Continuous bioreactor cultures

Continuous bioreactor cultures were run in a 0.5 L stirred tank bioreactor, Multifors 2 (Infors HT, Bottmingen-Basel, Switzerland). Temperature, pH, mixing, and airflow were fixed at 28 °C, pH 5.5, 1000 rpm, and 1.0 vvm. The cultures were conducted in YPG35 medium (g/L: yeast extract, 10, peptone, 20, glycerol, 35, maleic acid, 23.21) at a working volume of 0.3 L. The continuous feeding stage was initiated when glycerol was nearly completely consumed, highlighted by an increase in the pO2. During the continuous stage, the YPG35 medium was fed, and an equal volume of the culture was withdrawn. Pumps were set to maintain the dilution rate of approximately 0.10 [h-1]. Samples for the following transcriptomics analysis were typically collected > > 6 residence times (6 nV) (at ~ 100 h of the feeding stage), once the sFL level was stable. The collected samples were briefly centrifuged, and the biomass was washed in sterile saline solution. The supernatants and the washed biomass were stored at -80 °C for further analyses. All the cultures were conducted in at least two independent runs.

#### Sample preparation and RNAseq

Biomass samples to be analyzed via RNAseq were rapidly centrifuged, washed once in sterile saline solution, and resuspended in TRIzol (Ambion; Thermo Fisher Scientific); then, stored at -80 °C until shipment. The set of samples was forwarded to the Novogene facility for RNA isolation, quality control, library preparation, and sequencing on the Illumina platform, according to an in-house protocol. Briefly, the quality of RNA isolates was determined using BioAnalyzer (Agilent) capillary electrophoresis. mRNA was purified from total RNA using poly-T oligo-attached magnetic beads. After fragmentation, the first strand cDNA was synthesized using random hexamer primers, followed by the second strand cDNA synthesis. The library was ready after end repair, A-tailing, adapter ligation, size selection, amplification, and purification. Different libraries were pooled based on the effective concentration and targeted data amount, then subjected to Illumina sequencing.

#### RNAseq bioinformatics analysis pipeline

Raw data (raw reads .fastq) were processed through fastp software to remove reads containing adapters, reads containing poly-N, and low-quality reads from the raw data. Parameters Q20, Q30, and GC content were determined. HISAT2 (2.2.1; [54]) was used to map the clean reads to a reference genome (strain CLIB122 assembly ASM252v1) in a splicing-aware alignment mode. The mapped reads of each sample were assembled by StringTie (v2.2.3) [55] in a reference-based approach to assemble and quantify transcripts representing multiple splice variants for each gene locus. Gene expression level was calculated using featureCounts (2.0.6) [56]. FPKM (Fragments Per Kilobase of transcript sequence per Million base pairs sequenced) of each gene was calculated based on the length of the gene and the read count mapped to this gene, considering the sequencing depth. For each sequencing library, read counts were adjusted using the edgeR R package (4.0.16) [57] by scaling normalization factors to eliminate differences in sequencing depth between samples. Differential expression analysis between OE/KO/control strain samples was conducted using the DESeq2 R package (1.42.0) [58]. The resulting p-value was adjusted using Benjamini and Hochberg’s method to control the error discovery rate. The threshold of significant differential expression was set at padj < = 0.05. Gene Set Enrichment Analysis (GSEA) of differentially expressed genes (DEGs) was implemented by clusterProfiler (4.8.1) [59]. GO (Gene Ontology) terms (p-value < 0.05) were considered significantly enriched. Raw reads (fastq files) were deposited in NCBI SRA (Sequence Read Archive) under BioProject accession number: PRJNA1423913, and accompanying processed data (raw gene counts) were deposited in NCBI GEO (Gene Expression Omnibus), under accession number: GSE320446.

#### Differential exon usage analysis

Differential exon usage (DEU) was assessed using a DEXSeq framework [60, 61]. RNA-seq reads aligned to the reference genome were counted over exon bins. The relative exon usage between TF-modified strains and the control strain was modeled using generalized linear models. Exons were considered differentially used at an adjusted p-value < 0.05. Genes were classified as DEU-affected when at least one exon bin showed significant differential usage. The numbers of DEU exon bins and DEU genes were compared to the total number of annotated genes in *Y. lipolytica*, and the subset of multi-exon genes capable of alternative splicing. The statistical significance between TF-groups was calculated using Dunn’s post hoc test, with Benjamini-Hochberg correction for multiple testing.

## Results

### General notes

Based on the previous reports on the beneficial role of temperature downshift on the rProts secretion in *Y. lipolytica * [3, 51] the phenotyping was conducted under 22 °C and the standard 28 °C. Yet, the current data show that the difference in rProt synthesis between 22 °C vs. 28 °C was not statistically significant (p-value = 0.07, p-value = 0.13 for intracellular and extracellular scYFP fractions vs. the “control +”, respectively; p-value = 0.13, and p-value = 0.28 for extracellular levels of scYFP and scTlG vs. “control double +”, respectively) (Supplementary Material Figs. S3, S4, S5, S6, S7). Therefore, below we present the data acquired under one thermal condition, and full data showing TF-specific tendencies are given in the Supplementary Material (Figs. S3–S7.).

### Small, secretory protein production (scYFP)

**Fig. 2:**
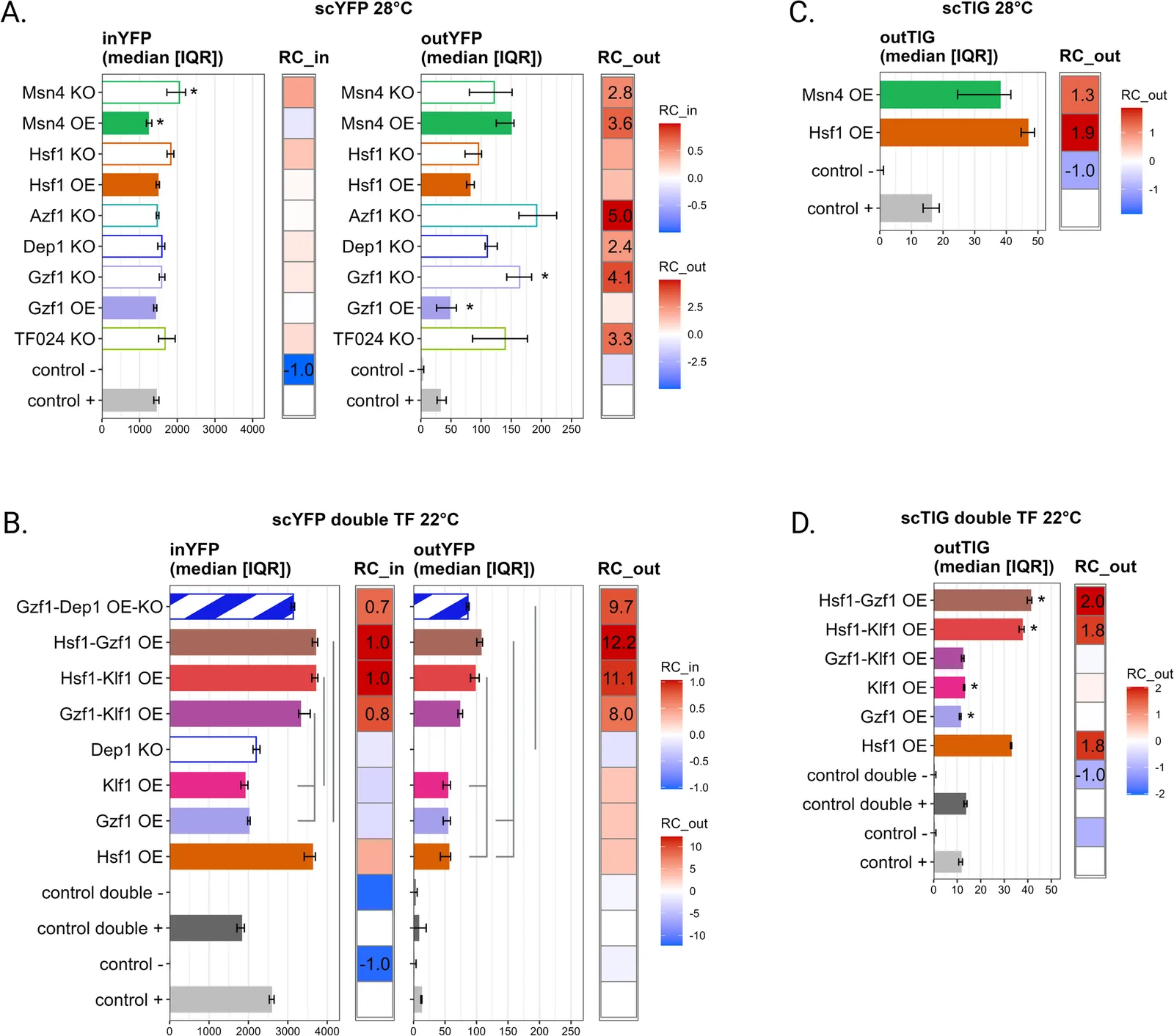
Levels of scYFP (**A**, **B**) or scTlG (**C**, **D**) produced by *Y. lipolytica* strains modified by OE (closed bars) and KO (open bars) of TFs, or a combination of two TFs OE (closed bars), or an OE and KO of TFs (striped bars). The type of modification is indicated on the y-axis. Bars show the median of normalized specific fluorescence (nsFL) or activity (nsGAU) determined in the biomass (inYFP) or in the culture supernatant (outYFP; nsGAU) at 48 h of culturing. Error bars show the interquartile range (IQR) based on at least 4 replicates. Six control strains were used as references to assess the change driven by TF-modification: (1) and (2) positive control (“control +“): Po1f derivative overexpressing solely (1) scYFP or (2) scTlG in JMP62-URA3ex vector, remaining auxotrophy complemented with an empty vector JMP62-LEU2ex vector, (3) negative control (“control –“): Po1f-derivative with both auxotrophies complemented with empty vectors; (4) and (5) double positive control (“control double +“): JMY8695 derivatives overexpressing 4. scYFP or 5. scTlG in JMP62-URA3ex vector, remaining auxotrophy complemented with an empty vector JMP62-LEU2ex vector and Lys5 solo cassette, (6) double negative control (“control double -“): JMY8695 derivatives with three auxotrophies complemented with empty vectors. Nr 1, 2, and 3 - control strains relevant for strains with a single TF modified, numbers 4, 5, and 6 - control strains relevant for strains with two TFs modified. Asterisks show statistical significance (padj < 0.05) of the nsFL values difference between the OE and KO strains, modified in the same TF. Grey lines show statistical significance (padj < 0.05) of the nsFL values difference between the OE strain (single modification) or the KO strain (single modification) and the double modification strains (OE-OE or OE-KO), modified in the same TFs. Asterisks show statistical significance (padj < 0.05) of the nsGAU values difference between the OE strain (single modification) and the double modification strains (OE-OE), modified in the same TFs. The heatmap next to the graph shows the relative change (RC) of a given metric (nsFL for in/outYFP; nsGAU for outTlG) in the given strain (signatures on the Y axis of the graphs) relative to the positive control strain (grey closed bar). The heatmap is color-coded according to the legend provided. Whenever the RC value was statistically significant, the value is indicated in the heatmap cell

Figure 2A shows the results of scYFP production in *Y. lipolytica* strains modified by both OE and KO of TFs (one TF modification per strain; both modifications for each TF). The level of the protein retained inside the cell (inYFP) and secreted outside the cell (outYFP) was analyzed. Foremost, we observed a consistent phenotype for the Gzf1-engineered strain (Fig. 2A). Strains lacking active Gzf1 displayed a significantly enhanced outer level of the rProt (vs. the Gzf1-OE and vs. the control). Of note, the inYFP levels remained unaffected, suggesting a specific action on the secretion.

We observed an interesting difference between KO and OE genotypes for the Msn4-modified strains. The Msn4-KO clearly triggered higher synthesis and accumulation of inYFP compared to its OE counterpart, but the outYFP parameter was significantly enhanced for both genotypes (Fig. 2A, inYFP). Surprisingly, none of the genotypes created in the *HSF1 locus* yielded a significant change in the scYFP synthesis.

On the other hand, striking results were observed for the Azf1-KO strain, which significantly promoted secretion of the rProt (RC of 5.0), maintaining the intracellular YFP levels comparable to the control. A highly similar effect was observed for the TF024-deletant. They both displayed a specific, secretion-promoting phenotype.

None of the modifications significantly modulated the intracellular levels of the reporter protein vs. the control. We were specifically interested in the outcomes of the Dep1-KO strain, as it previously brought the highest rProt synthesis-promoting effect (with a small fluorescent protein targeted for intracellular localization) [12]. Here, with an rProt targeted for secretion, deletion of *DEP1* did not enhance rProt levels.

In addition to modifying a single TF per strain, several selected engineering approaches were implemented in combination. These were either double co-overexpression (OE of two TFs and the rProt) or single co-overexpression in the TF-KO background (OE of TF and rProt, KO of *DEP1*). The previous results [12] guided the selection of the combinations. Figure 2B depicts the levels of the intracellular and secreted fraction of scYFP reporter by the double-TF-modified strains, in comparison with the single-TF modifications and the controls.

When implemented solo, OE of Klf1, Gzf1, and Hsf1 triggered insignificant enhancement in the outYFP levels. However, when any of the OE genotypes were combined, the rProt amounts surpassed the minor beneficial effects of individual modifications, yielding the highest RC values in outYFP reported in this study (8.0 to 12.2; Fig. 2B). The intracellular amounts of the rProt were also significantly higher. Interestingly, a combination of two modifications that individually yielded mediocre results (Gzf1-OE and Dep1-KO) resulted in a highly beneficial phenotype (outYFP RC 9.7). While for the control strains, the temperature downshift had no significant effect on the amounts of rProt produced, for the double TFs modification strains, the temperature downshift was a prerequisite for the revelation of the phenotypes.

### Production of UPR-inducing secretory protein (scTlG)

Figure 2C presents the results on the extracellular activity of a second rProt used in this study – fungal glucoamylase, scTlG. The rProt was selected based on its previously reported strong propensity for the UPR induction in *Y. lipolytica* [17]. The extracellular activity was used as a proxy for the protein’s extracellular amounts and expressed in normalized values per biomass unit and the control strain (nsGAU). It is, though, clear that the extracellular activity does not perfectly mirror the rProt production level driven by the TF-modified system, as the other factors like folding, glycosylation, secretion, etc., impact this parameter. Moreover, the intracellular amounts of scTlG were not studied, as it was shown previously that until fully maturated and secreted, the enzyme remains inactive [53, 62, 63].

The most striking observation was a two-fold increase in the scTlG measure (RC 1.9) in the strain co-overexpressing Hsf1 (Fig. 2C). A similar promoting effect was observed for the Msn4-OE strain (RC 1.3). In good agreement with what was observed for the Hsf1-OE solo modification, any Hsf1-coupled overexpression, either combined with Gzf1 or Klf1, yielded a significant enhancement in scTlG production levels (Fig. 2D). The effect was clearly driven by the Hsf1 upregulation, as the double overexpression strain (Hsf1-Gzf1 OE) produced significantly more scTlG (RC 2.0) than the solo Gzf1-OE counterpart (not significant at *p* < 0.05), but not Hsf1-OE solo (RC 1.8). Moreover, when the double overexpression combination was composed of Gzf1 and Klf1, the resulting scTlG levels were comparable to the control (not significant at *p* < 0.05).

### Insight into transcriptomes of selected TFs – Azf1 and Dep1

### General information

The next goal was to get insight into the molecular background of Azf1’s beneficial role in the secretory rProt production. Azf1, similarly to Dep1, was previously identified as a potent repressor of intracellular rProt (inRFP) synthesis [12] (Fig. 1). Their overexpression caused a significant decrease in RFP production [12]. However, with the secretory rProt studied here, only the Azf1-KO genotype yielded an enhanced secretor phenotype. To better understand the Azf1 regulome, the Dep1 transcriptome was also sequenced, mainly for comparative purposes. Because Dep1-KO displayed its beneficial action solely towards inRFP and not scYFP (Fig. 2A), we decided to analyze the regulome of this advantageous phenotype. For the sake of consistency and to facilitate valid comparisons, the Azf1 regulome was also studied in the inRFP-producing background (Azf1 was operable for both inRFP and scYFP; Fig. S8 and Fig. 2; respectively). So the below-described regulomes represent TFs’ transcriptomes in the background of intracellular rProt production. To the best of our knowledge, transcriptomes of Azf1 and Dep1 have not been published to date (available transcriptomes reviewed recently [64]).

The amounts of the rProt determined in the biomass of the TF-modified strains in a semi-steady state were as expected (Fig. S8). The KO strains displayed 2- to 3-fold higher nsFL yields than the OE and the control strains. A stable, genotype-driven phenotype expressed at comparable levels in both the small-scale batch cultures and the continuous bioreactor cultures holds a promise for the investigation of the transcriptomes underlying the trait-of-interest.

**Fig. 3:**
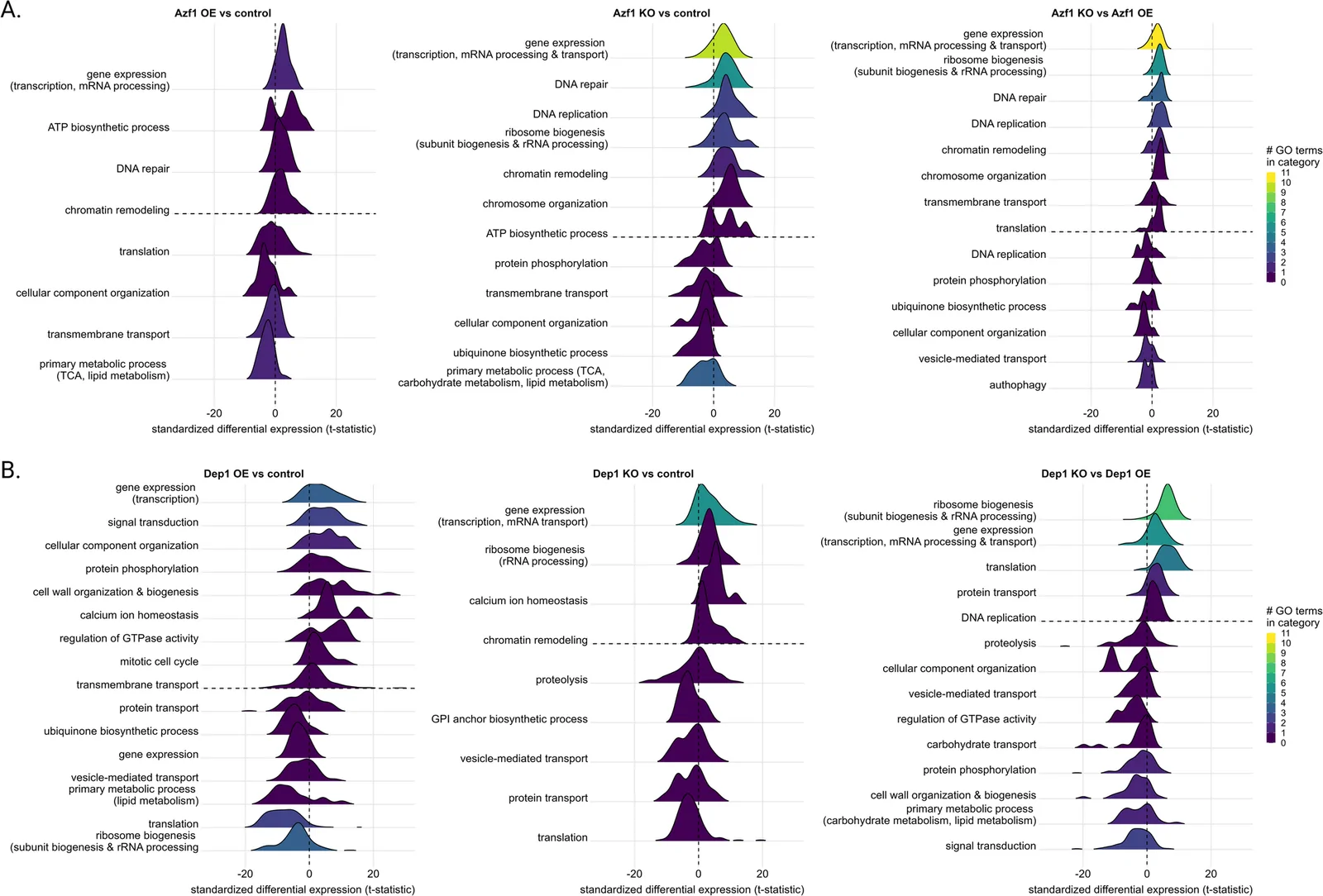
GO terms enriched in transcriptomes of **A** Azf1-OE and Azf1-KO **B** Dep1-OE and Dep1-KO strains. The GSEA was conducted on DEGs between the TF-modified strains and the control strain, resulting in a list of differentially enriched GO terms, each assigned a specific NES value (full list in SM). The deregulomes were contrasted Azf1-OE vs. control, Azf1-KO vs. control, and Azf1-KO vs. Azf1-OE (**A**), and Dep1-OE vs. control, Dep1-KO vs. control, and Dep1-KO vs. Dep1-OE. Initial extensive sets of differentially enriched GO terms (SM) were further processed to organize the lower-level GO terms into higher-level GO terms by their similarity. The ridgeplots present a list of biological processes GO terms differentially enriched in a given transcriptome vs. the comparison transcriptome. Each ridgeplot represents a set of genes with a corresponding t-statistic (standardized differential expression: level of deregulation vs. padj) classified to a given GO term overrepresented in a given transcriptome vs. comparison partner. The GO terms were classified from the most upregulated with the best statistics (top) to the least upregulated (middle; above the dashed line), and from the least downregulated with the weakest statistics (middle; below the dashed line) to the most downregulated with the best statistics (bottom). Centrally located dashed axis differentiates the genes into upregulated (right side) and downregulated (left side). The ridgeplots are color-coded according to provided legends, illustrating the number of lower-level GO terms accounting for to higher level GO terms indicated on the Y axis of the graph

Omics data were analyzed as described in the Materials and Methods section. We primarily focused on gene set enrichment analysis (GSEA) to characterize the general molecular landscape shaped by the TFs, to assess their usefulness as global transcriptional machinery engineering tools. The significance of the GO term enrichment was determined in comparison to: (i) the control strain, and (ii) the inverted TF-engineered counterpart. Results of GSEA, limited to the higher-level of GO ontology terms, are presented in Fig. 3A, B. A full list of GO terms significantly deregulated is given in Supplementary Material (SM).

#### Azf1 – mRNA processing, splicing, chromatin, ribosomes, carbon, and signaling

When compared to the control strain, the primary functional axis activated in the Azf1-OE strains was associated with RNA processing and splicing (mRNA splicing, catalytic step 2 spliceosome, U1/U4/U6/U5, complexes, Prp19 complex; normalized enrichment score (NES) values 2.35, 2.23, 1.93, 1.81, 1.74; “gene expression” in Fig. 3A; Table 1 in SM). Consistently, the central hub for RNA polymerase II transcription, the mediator complex (NES 1.92), and transcription by Poly II (NES 1.99) were both significantly enriched. The increased accessibility of DNA for transcription was highlighted by upregulation of chromatin remodeling (NES 1.76–1.74). The profile is completed by downregulation in mitochondrial activity (mitochondrion, iron-sulfur cluster assembly, tricarboxylic acid cycle, FMN binding; NES < -1.75; “primary metabolic process”), translation and ribosomes, ribosome binding (NES − 1.55, -1.69, -2.0), as well as membrane-localized processes and transmembrane transportation (NES − 1.74). Changes in cellular component organization (Fig. 3A) relate to all – cytosol, mitochondrion, and membrane (Table 1 in SM).

Contrasting the KO-Azf1 regulome with the control, we observed positive, strong enrichment represented by multiple terms related to “gene expression” (Fig. 3A; Table 2 in SM) (DNA binding (NES 2.89), RNA polymerase II, core complex, DNA-templated transcription, transcription by RNA polymerase II transcription elongation by RNA polymerase II (NES 1.95–2.03), RNA binding, mRNA export (NES 2.06–2.12), and splicing (NES 2.54–2.51)). Another enriched functional axis was represented by nucleolus-localized processes (NES 2.72) and ribosome biogenesis (ribosome biogenesis, preribosome, large subunit precursor, rRNA processing, RNA polymerase I complex, small-subunit processome, NES 1.79–2.15). Furthermore, positive enrichment in term “mitotic spindle” (NES 2.03) together with “DNA replication” (NES 2.46; DNA replication initiation, DNA replication origin binding, DNA helicase activity, DNA directed DNA polymerase activity), “DNA repair” (NES 2.26; DSB repair, BIR, telomere maintenance), and “chromatin remodeling” (NES 2.24), highlight an enhanced proliferative programme (DNA repair and replication in Fig. 3A). Amongst the significantly enriched functionalities, massive downregulation in catabolism and energy-gaining was revealed within processes localized to all cytosol, mitochondria, and peroxisomes (e.g., glycolytic process, carbohydrate metabolic process, formate dehydrogenase (NAD+) activity, 4 iron, 4 sulfur cluster binding, fatty acid beta-oxidation, tricarboxylic acid cycle, peroxisome, mitochondrion, ubiquinone biosynthetic process etc.; for NES values see Table 2 in SM). These are also reflected in the cellular component organization term in Fig. 3A. A slight downregulation of protein phosphorylation suggests quenching of the signaling network (Fig. 3A).

Counterintuitively, the enrichment in several key biological processes, like gene expression and splicing, chromatin remodeling, or mitochondrial processes, was shared and deregulated in the same direction in Azf1-OE and Azf1-KO genotypes. Therefore, to investigate the differentiating molecular background behind the beneficial Azf1-KO phenotype, we contrasted the regulomes displayed by Azf1-KO vs. Azf1-OE genotypes (Fig. 3A; Table 3 in SM). The principal signatures that emerged were associated with transcription, splicing, translation, and ribosome biogenesis (including i.a., nucleolus, rRNA processing, preribosome, translational initiation, translation initiation factor activity, mRNA binding, DNA-directed RNA polymerase activity, RNA polymerase I/II/III complex, mRNA splicing via spliceosome, RNA binding, RNA helicase activity etc.; NES > 2.0 up to 3.08). These processes were significantly upregulated and enriched in the Azf1-KO regulome compared to the Azf1-OE. Likewise, the Azf1-KO regulome was enriched in “chromatin remodeling” (histone binding, chromatin binding, chromatin, nucleosome binding, ATP-dependent chromatin remodeler activity; NES all > 1.5) and “DNA repair” terms (DNA repair, mismatch repair, damaged DNA binding; NES 2.04) when compared to the Azf1-OE. On the other hand, contrasting the KO vs. OE Azf1 transcriptomes highlighted a significant downregulation of the GO terms related to mitochondrial activity (mitochondrial respiratory chain complex IV assembly, mitochondrial inner membrane/intermembrane space, mitochondrion, ubiquinone biosynthesis, FAD binding, oxidoreductase activity; NES < -1.7); represented by ubiquinone biosynthetic process, cellular component organization, and autophagy in Fig. 3A. Surprisingly, a wide range of GO terms related to vesicular transportation were also downregulated in Azf1-KO vs. OE (SNARE complex, SNAP receptor activity, vesicle fusion, vesicle-mediated transport, microtubule cytoskeleton; NES < -1.8; Table 3 in SM). The contrast KO vs. OE pointed to Azf1’s role in restructuring (signaling and executing) cell morphology. Indeed, when verified microscopically, the Azf1-OE strain displayed enhanced filamentation (Fig. S9). Of note, the key impact of cell wall condition on rProts secretion in yeast has been recently elegantly proved and implemented in engineering strategies aiming at enhanced secretory rProts production [65, 66].

**Fig. 4:**
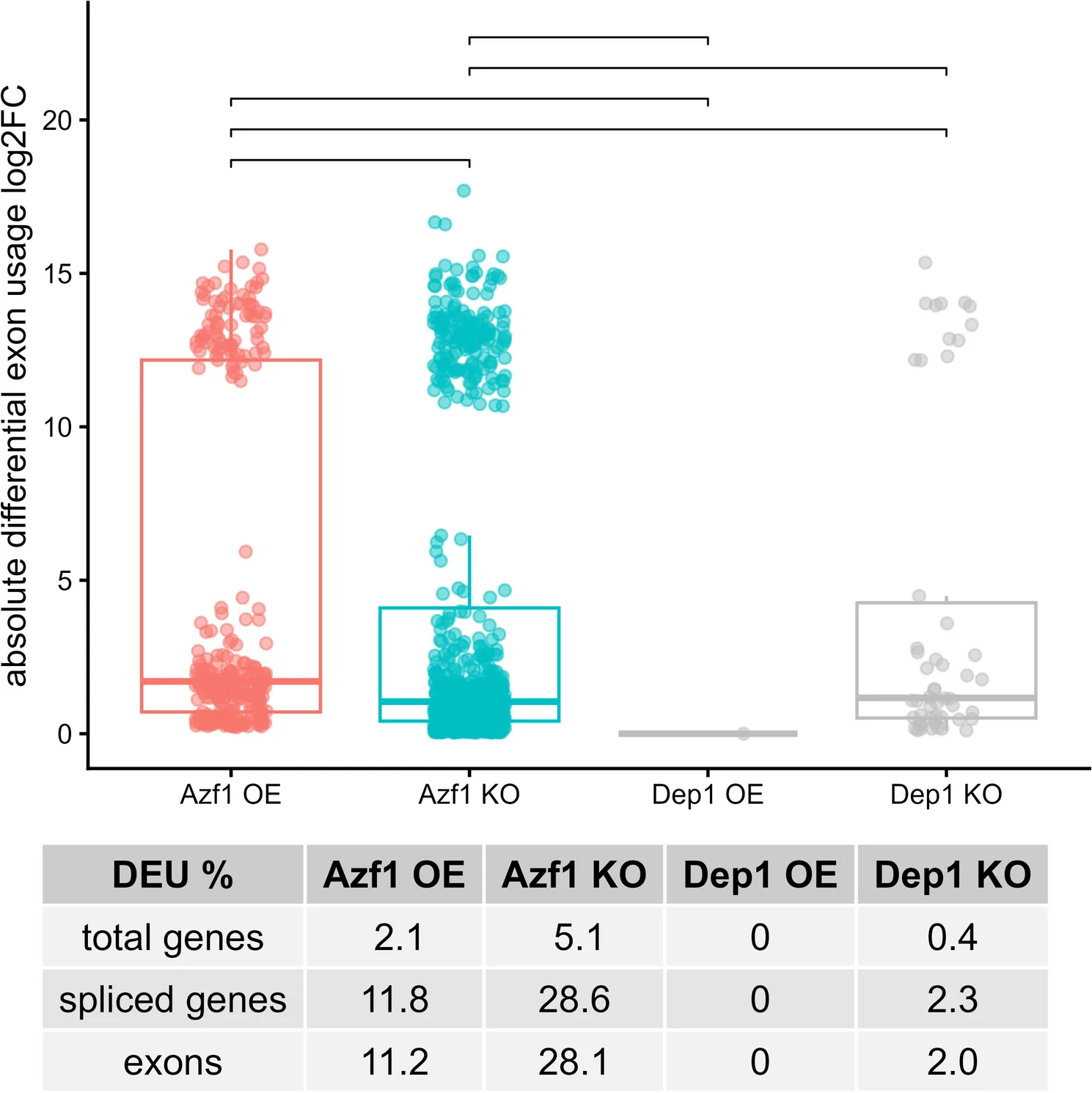
Global differential exon usage across the TF-modified strains’ transcriptomes. Boxplots show the distribution of absolute log₂ fold changes for statistically significant differentially used exon bins, relative to the control strain. Statistically significant differences between strain groups are indicated in the plot. The inset table summarizes: the percentage of genes containing ≥ 1 DEU exon relative to: (i) all annotated *Y. lipolytica* genes and (ii) multi-exon genes capable of alternative splicing; and (iii) the percentage of DEU exon bins relative to the total number of exon bins in multi-exon genes

Considering that RNA processing and splicing were the primary functional axes activated in both the Azf1-OE and Azf1-KO strains, to understand the difference, we decided to run differential exon usage analysis on the two transcriptomes (Fig. 4; Table 4 A, B, C, D,E in SM).

The adopted DEU analysis is independent of gene expression levels and refers solely to changes in exon usage across all genes in the transcriptome. The Dep1-engineered strains were included as a control to set a baseline for the changes in the Azf1-modified strains. Both Azf1 KO and OE strains exhibited a substantial fraction of genes and exon bins (~ 12% for Azf1-OE, ~ 29% for Azf1-KO) with differential exon usage relative to the control strain (Fig. 4, table inset). This observation is consistent with the GSEA results, in which the term “mRNA splicing, via spliceosome” was significantly enriched within the broader category of “gene expression” (Fig. 3A; Table 3 in SM). GO term classification indicated that the differentially-spliced genes were involved in ribosome biogenesis, rescue, translation, nucleotide synthesis, cellular respiration, and vesicular transportation for Azf1-KO (Table 4E in SM). For the Azf1-OE strain, the scope of biological processes was relatively narrow, and the differentially-spliced genes mainly represented cytoplasmic translation and gene expression (Table 4E in SM).

#### Dep1 – ribosomes and translation in focus

GSEA run on datasets Dep1-OE and Dep1-KO vs. control displayed several major biological processes enriched in the Dep1-modified strains (Fig. 3B; Tables 5 and 6 in SM). Foremost, we observed a strong (NES up to 2.6 in Dep1-OE, and 2.3 in Dep1-KO) signal from the gene expression-related terms (RNA Pol II cis-regulatory binding, DNA-binding TF activity, and regulation of transcription). Terms chromatin/chromatin remodeling and ATP-dependent chromatin remodeler activity (NES 1.95 to 2.23) were upregulated solely in the KO strain (vs. control); suggesting Dep1’s involvement in chromatin stability and DNA accessibility for transcription. Translation, nucleolus, rRNA processing, and ribosome structural elements (NES from − 2.08 down to -3.5) were massively deregulated in the Dep1 OE and KO strains. Yet, a prominent difference was found here; all the terms related to ribosomal structural elements or function were downregulated in the Dep1-OE, and upregulated in the Dep1-KO strain (Fig. 3B). Terms related to signal transduction, like serine/threonine kinase, phosphorylation, signal transduction/GTPase-mediated signaling, and intracellular calcium ion homeostasis (NES 1.0–2.1) were all enriched and upregulated in the Dep1-OE strain, suggesting Dep1’s role in global regulation of the cell’s state. Consistently, both transcriptomes (OE and KO) were enriched in microtubule cytoskeleton/actin filament organization (up in Dep1-OE, down in Dep1-KO), which, together with signaling and cell wall organization (the latter specific to Dep1 OE regulome), suggests that Dep1 acts as a global regulator of cellular architecture and cellular homeostasis. As in the case of Azf1, the reversing perturbations, either OE or KO, happened to deregulate several functional axes in the same direction (not valid for ribosome biogenesis).

Therefore, to get a deeper insight into the molecular background behind the beneficial phenotype driven by the Dep1 KO genotype, we contrasted the OE and the KO Dep1 regulomes. Strikingly, the Dep1-KO transcriptome was dominated by terms classified to “ribosomes and cytoplasmic translation” (Fig. 3B; for multiple terms NES > 3.0; Table 7 in SM): structural constituent of ribosome, ribosome, cytosolic large/small ribosomal subunit, cytoplasmic translation, translation, translation initiation factor activity, eIF3 complex. In addition, terms related to nucleolus and rRNA processing (small-subunit processome, 90 S preribosome, preribosome large subunit precursor, ribosomal large/small subunit assembly, ribosomal large subunit biogenesis, ribosome biogenesis) further strengthen this functional axis. In line, the activity of RNA polymerase I/II, rRNA binding, as well as protein import/mRNA export into/from the nucleus, were also over-represented in the Dep1-KO regulome with highly positive NES (Table 7 in SM). The very high NES values (up to 3.6), very low padj, and the extremely wide scope of the consistent GO terms altogether suggest that the Dep1 deletion fires up expression of genes involved in ribosome biogenesis and cytoplasmic translation. The mitochondrial translation was also upregulated, as highlighted by mitochondrial translation, as well as protein import into the mitochondrial matrix (NES ~ 2.0).

#### Azf1 vs. Dep1 – differentiating vesicular transportation

**Fig. 5:**
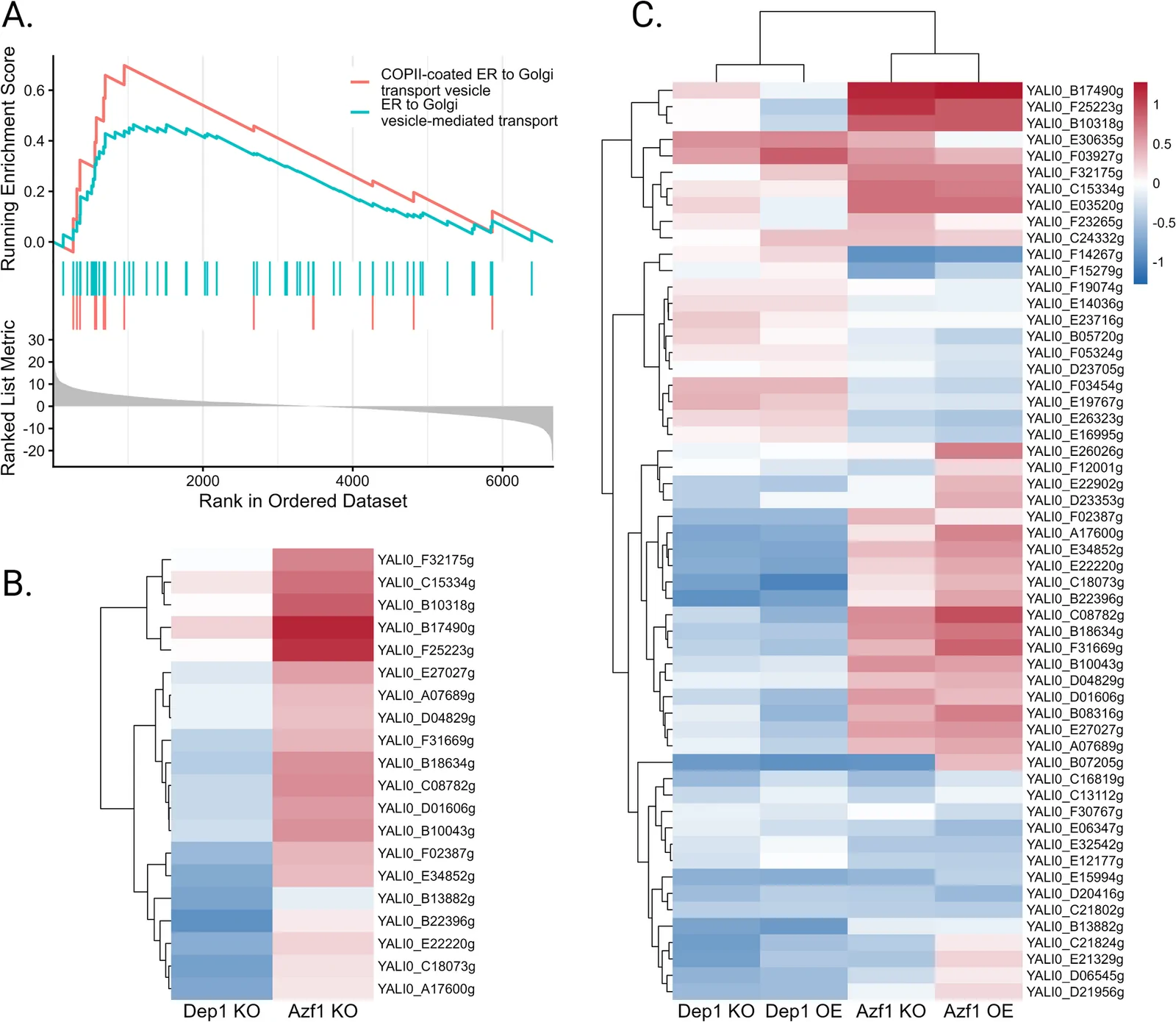
Vesicle-mediated transportation-focused GSEA. **A** GSEA plot highlighting two GO terms related to ER-Golgi vesicle transport that distinguish Azf1 KO from Dep1 KO: a cellular component term (orange), a biological process term (cyan). The top panel shows the running enrichment score (ES), the middle panel visualizes distribution of genes belonging to the GO terms across the ranked list of genes deregulated in Azf1 KO vs. Dep1 KO, and the bottom panel displays the ranked gene list ordered by t-statistic. **B** Heatmap of the core enrichment genes (*n* = 20) driving the positive ES/NES values of both GO terms. Gene expression is shown as logFC in Azf1 KO and Dep1 KO relative to control. **C** Expression of DEGs from GO term “ER to Golgi vesicle-mediated transport” (*n* = 56) across Azf1 and Dep1 OE and KO strains. Heatmap scaling is shared between panels (**B**–**C**), and clustering of genes and strains is based on Euclidean distance. Identification of the closest homologue and its name in *S. cerevisiae* is provided in Tab.S9

The GO term “*vesicle-mediated transport*” was downregulated in KO-OE comparisons for both Azf1 and Dep1. However, at the phenotype level, the Azf1-KO drove significantly higher scYFP secretion than the Dep1-KO (Fig. 2A). This contradiction prompted a direct GSEA comparison between Azf1-KO and Dep1-KO (Fig. 5; Table 8 in SM). Strikingly, two significantly enriched GO terms related to ER-Golgi vesicle trafficking (“*COPII-coated ER to Golgi transport vesicle*”; cellular component, 14 genes, NES 1.88; “*ER to Golgi vesicle-mediated transport*”; biological process, 56 genes, encompassing all genes from CC, NES 1.77) were significantly upregulated in Azf1-KO relative to Dep1-KO. Figure 5B illustrates the differential expression profile of 20 core enrichment genes that were the principal contributors to the positive enrichment signal. They were all significantly upregulated in the Azf1-KO.

To contextualize vesicle-mediated transport regulation across TF perturbations, we present expression profiles of all 56 genes within these GO terms across Azf1 and Dep1 OE and KO strains (Fig. 5C; Table 9 in SM). Vesicle-mediated transport was not enriched in OE vs. KO contrasts for either TF, indicating that most deregulation occurs in a consistent direction within each TF background rather than between OE and KO states. Clustering analysis resolved four principal expression patterns (Fig. 5C): genes upregulated in all strains relative to control (cluster 1); upregulated in Dep1-modified strains but downregulated in Azf1-modified strains (cluster 2); genes showing opposite regulation between Azf1 and Dep1, including most of the core enrichment genes from Fig. 5B. (cluster 3); genes broadly downregulated across strains, with a minor subset upregulated in Azf1-OE (cluster 4). Functional classification of these clusters using PANTHER identified cluster-specific biological themes within the ER-Golgi trafficking pathway (Table 10 in SM).

Clusters 1 and 3, both upregulated in the Azf1 background, were primarily associated with generic ER-to-Golgi vesicle-mediated transport. No distinct subprocesses uniquely separated these two clusters (they were separated based on Dep1-driven differential response). However, both showed enrichment for components related to vesicle tethering, including the TRAPP complex and vesicle tethering complex, pointing to vesicle targeting and docking at the Golgi rather than vesicle formation at the ER (refer to Table 10 in SM). Greater functional specificity emerged in the clusters downregulated in Azf1 (clusters 2 and 4). Cluster 2 was enriched for COPII-coated vesicle cargo loading and COPII-coated vesicle budding, suggesting that this cluster primarily captures transcriptional variation in vesicle formation at the ER exit site. Yet, in the cellular component area, COPI- vesicle coat-related terms were abundantly represented, suggesting that the retrograde Golgi-to-ER route is enriched. Consistently, cluster 4 emphasized processes related to Golgi-to-ER retrograde transport and ER protein retention (reflected in cluster-specific enrichment of processes such as protein retention in ER lumen, maintenance of protein localization in ER, retrograde vesicle-mediated transport, Golgi to ER), with a small SNARE-associated signal: enrichment of the SNARE complex (cellular component) and SNAP receptor activity (molecular function).

## Discussion

While a wide set of TF-modified strains was analyzed here, only several displayed consistent, unbiased phenotypes that could be biologically interpreted with the current data. Among the most interesting observations, we found a specific action of two general stress response TFs – Msn4 and Hsf1. They elicited their beneficial effects solely towards the highly glycosylated, UPR-inducing scTlG, and not the “non-problematic” scYFP. Previously, we evidenced that overproduction of scYFP did not induce any signs of UPR displayed at the transcriptional level, in contrast to scTlG or inYFP [17]. Apart from the transcriptional profiles, the “difficult” and “simple” classification was attributed based on the rProts amounts: scYFP was produced at high levels, over 10-fold higher than inYFP. Similarly, the molar levels of TlG were low when compared to the other enzymatic reporter used in that previous study. Therefore, to our interpretation, the scYFP synthesis and secretion were processed unbottlenecked, so that the overrepresentation of Msn4 or Hsf1 did not elicit any significant impact. When the rProt synthesis was associated with massive cellular stress, scTlG [17] the augmenting action of the stress response TFs was displayed (Fig. 2C). Hsf1 was also the major driver of scTlG production enhancement in double scTlG mutants (strains overexpressing two TFs). Whenever Hsf1-OE was inflicted, an increase in outTlG levels occurred (Fig. 2D). An impact of Hsf1-OE on the “simple” rProt, scYFP, could be seen, but only when double TFs OE was introduced (Fig. 2B). Such an outcome suggests that an increased burden of translational machinery is needed to reveal the Msn4/Hsf1-driven response.

Based on the insight from transcriptomics data describing Azf1 and Dep1 regulomes, we carefully propose an interesting hypothesis on an important interplay between ribosomal-translational machinery and vesicular transportation axes. That observation was facilitated by contrasting the regulomes of Azf1 (promoting synthesis and secretion) against Dep1 (promoting synthesis only). Both the biological processes (localized to specific cellular compartments) were co-regulated in the Azf1-KO-driven phenotype, but the vesicular transportation axis was missing from the Dep1-driven regulome.

In detail, the Azf1-engineering induced a massive response in transcription and splicing (Fig. 3A; Tables 1, 2, 3 and 4 A, B, C, D, E in SM). What specifically differentiated the KO from the OE Azf1 regulome was a strong upregulation in the ribosome biogenesis and translation; a wide set of related GO terms was upregulated in the KO, and was downregulated in the OE genotype (Table 3 in SM). Moreover, the data presented in Fig. 5, demonstrate upregulation in genes involved in vesicle-mediated transportation (cluster 1 and cluster 3; enriched in ER-to-Golgi vesicle-mediated transport) that could potentially contribute to the highly potent secretor phenotype displayed by Azf1-KO. If confirmed in experiments directly gauging these processes, the Azf1-KO would confer a “dual gain” only for the secretory rProt. A recent article reported successful strategies focused on the early secretion pathways engineering *K. phaffii*, that, when combined with membrane expansion, modulation of ER-associated degradation (ERAD), and vesicular transport enhancement, led to over 6-fold improved secretion of the target rProt [67]. Strikingly, the results of the DEU analysis showed that splicing events were mainly executed on ribosome biogenesis, translation, energy gaining, and vesicular transportation for Azf1-KO genes (Fig. 4; Table 4E in SM). Previously [46, 68], showed that the *Y. lipolytica* genome is enriched in introns compared to a model yeast species, suggesting an important role of alternative splicing in transcriptional regulation. The differential exon usage analyses run here showed that, indeed, between ~ 12% to ~ 29% of exons were used differentially in Azf1-OE and Azf1-KO regulomes, respectively. Considering this, the results of differential gene expression (Fig. 3A, B; Tables 1, 2, 3, 5, 6 and 7 in SM) and the alternative-splicing (Fig. 4; Table 4 A, B, C, D, E in SM) are consistent with each other and complementary, demonstrating the concerted action of transcriptional regulation and alternative splicing contributing to the final TF-driven transcriptome. This leads to an interesting, yet still to be experimentally verified, hypothesis that Azf1-KO genotype executed its rProt synthesis-promoting effect using an alternatively-spliced regulome. It also remains to be settled whether the alternatively spliced gene variants directly contributed to enhanced rProt synthesis/secretion.

Our previous results revealed that OE of Dep1 (and Azf1) led to a drop in inRFP levels, but its KO led to an inverted phenotype [12, 14] (Fig. 1). In fact, Dep1-KO was one of the most prominent rProt accumulation phenotypes identified previously [12]. Surprisingly, in the present study, the inYFP levels were not enhanced when the rProt was targeted for secretion (Fig. 2A). Even more puzzling was the effect of Dep1 deletion on outYFP levels (Fig. 2A). To understand the difference between inRFP vs. scYFP (and contrast with the Azf1’s operable phenotype), we set out to determine Dep1’s regulome. Translational machinery, its structural constituents, and effectors were the core elements of the Dep1-KO transcriptome. Based on the contrast between Dep1 KO and OE, the Dep1-OE regulome was portrayed as relieved from excessive investment in the translational apparatus, attending to cell wall restructuring, and, potentially, accumulation of carbon reserves. The observed propensity for filamentation by the Dep1-OE strain vs. the control strain (Fig. S9) confirms that statement at the phenotypic level. What we discovered was that all the terms related to ribosomal structural elements or operation were downregulated in the Dep1-OE and upregulated in the Dep1-KO strain (Fig. 3; Table 7 in SM). It supports our observation on the beneficial role of Dep1-KO on rProt synthesis when intracellular. Yet, it does not explain the lack of such a prominent outcome towards the secretory rProt. In this aspect, the contrast Dep1 KO vs. OE (Fig. 3; Table 7 in SM) showed that the genotype advantageous for rProt synthesis (Dep1-KO) is scarce in “vesicle-mediated transportation”, “signal transduction”, and “cytoskeleton organization” terms, suggesting that the deletion of Dep1 downregulates genes in vesicle-mediated transportation (stronger compared to what was recorded for Dep1-OE), potentially disabling efficient scYFP traverse. Then, to explain the Azf1-KO “super-secretor” phenotype, we ran a more detailed analysis on vesicle-mediated transportation for both Azf1 and Dep1 (Fig. 5; Tables 8 and 9 in SM). That contrast revealed genes in cluster 3, that were differentially expressed in Azf1-KO and Dep1-KO regulomes (Fig. 5; Table 8 in SM). To our interpretation, these genes represent plausible candidates contributing to uniquely enhanced secretory capacity in *Y. lipolytica* Azf1-KO; their downregulation potentially disabled the same effect in Dep1-KO, which had enhanced translational capacity.

## Conclusions

In conclusion, we demonstrated that the known general stress response TFs, Msn4 and Hsf1, elicited their rProt-promoting role only towards “challenging” rProt - scTlG (inducing metabolic stress when overexpressed). Their overrepresentation did not affect the levels of “simple” rProt, scYFP. We suggest that an additional impact of cellular stress is required to activate the beneficial role. Whether a rProt is challenging is to be defined, e.g., based on transcriptomes elicited by their overexpression (as in our case). Deletion of the previously identified universal repressors of intracellular rProt synthesis, Dep1 and Azf1, was associated with massive upregulation of ribosome biogenesis and cytoplasmic translation. However, only Azf1-KO’s regulome was enriched in vesicle-mediated transport components, which most probably led to significantly enhanced secretory scYFP production. We suggest that the Azf1-KO conferred a “dual gain” for the secretory rProt, for which the two biological processes (ribosomes and transportation) were beneficially deregulated. In the case of Dep1, ribosome biogenesis and translation were upregulated in the deletant. However, deeper insight into Dep1’s transcriptome revealed that the regulome is accompanied by limited vesicle-mediated transportation. Yet, such a mechanistic explanation requires further in-depth molecular studies.

This study deepens the knowledge about TFs in *Y. lipolytica*, providing data-driven hypotheses to be further tested with the other secretory rProts and with different experimental approaches.

## Supplementary Information

Below is the link to the electronic supplementary material.

Supplementary Material 1.

Supplementary Material 2. RNAseq dataset. Tables 1-8, GSEA analyses between the indicated datasets. Table 9, DEGs classified to GO term vesicular transportation; Table 10, Panther themes enriched in the 4 clusters differentiated within “vesicular transportation”-classified genes (refer to Fig.8.C).

## Data Availability

All the data are available directly in the manuscript or the Supplementary Materials. Raw RNAseq datasets were deposited in the NCBI-SRA database under BioProject: PRJNA1423913, GEO Accession: GSE320446.
