# Supplementary material for: Transcriptional reprogramming of the stress response and translational-secretory machinery enhances recombinant secreted protein production in Yarrowia lipolytica
Source: Microb Cell Fact. 2026 Jul 20;25:212. doi: 10.1186/s12934-026-03062-2 (PMC13670810; doi:10.1186/s12934-026-03062-2)
Supplement: Supplementary file 1 [file 12934_2026_3062_MOESM1_ESM.docx]

**Supplementary data**

**Table S1**. List of *E. coli* strains used in this study

| ECB number | Parental strain (background) | Vector | Gene / modification |
| --- | --- | --- | --- |
| ECB_32 | JM109 | JME3265 | JMP62-LYS55ex |
| ECB_33 | JM109 | JMP62 | JMP62-URA3 |
| ECB_34 | JM109 | JMP62 | JMP62-Leu2 |
| ECB_98 | JM109 | pV2-EcoRI NotI | ARMup TF128 - NATr-ARMdwnTF128 |
| ECB_99 | JM109 | pV2-EcoRI NotI | ARMup EUF1 - NATr-ARMdwn EUF1 |
| ECB_102 | JM109 | pV2-EcoRI NotI | ARMup TF124 - NATr-ARMdwnTF124 |
| ECB_103 | JM109 | pV2 (GGE140) EcoRI-NotI | ARMup HAP1 - NATr-ARMdwn HAP1 |
| ECB_190 | DH5α | JMP62_Leu2ex-pTEF | HAC1_codon optimized, intronless, ER removed |
| ECB_191 | DH5α | JMP62_Leu2ex-pTEF | SFP1_codon optimized, intronless, ER removed |
| ECB_192 | DH5α | JMP62_Leu2ex-pTEF | KLF1_codon optimized, intronless, ER removed |
| ECB_193 | DH5α | JMP62_Leu2ex-pTEF | HSF1_codon optimized, intronless, ER removed |
| ECB_194 | DH5α | JMP62_Leu2ex-pTEF | HAP1_codon optimized, intronless, ER removed |
| ECB_195 | DH5α | JMP62_Leu2ex-pTEF | GZF1_codon optimized, intronless, ER removed |
| ECB_198 | DH5α | JMP62_Leu2ex-pTEF | MHY1_codon optimized, intronless, ER removed |
| ECB_238 | DH5α | JMP62_Leu2ex-pTEF | Yas1 |
| ECB_241 | DH5α | JMP62_Leu2ex-pTEF | Mns4 |
| ECB_252 | DH5α | JMP62-LYS5 | KLF-LYS5 |
| ECB_254 | DH5α | JMP62-LYS5 | GZF-LYS5 |
| ECB_256 | DH5α | JMP62-LYS5 | HSF-LYS5 |
| ECB_266 | DH5α | pV2 (GGE140) EcoRI-NotI | ArmUP Cat8 - Nat - ArmDN Cat8 |
| ECB_269 | DH5α | pV2 (GGE140) EcoRI-NotI | ArmUP Yas1 - Nat - ArmDN Yas1 |
| ECB_272 | DH5α | pV2 (GGE140) EcoRI-NotI | ArmUP Msn4 - Nat - ArmDN Msn4 |
| ECB_277 | DH5α | pV2 (GGE140) EcoRI-NotI | ArmUP Mhy1 - Nat - ArmDN Mhy1 |
| ECB_279 | DH5α | pV2 (GGE140) EcoRI-NotI | ArmUP Sfp1 - Nat - ArmDN Sfp1 |
| ECB_288 | DH5α | pV2 (GGE140) EcoRI-NotI | ArmUP TF024 - Nat - ArmDN TF024 |
| ECB_289 | DH5α | pV2 (GGE140) EcoRI-NotI | ArmUP BRG1 - Nat - ArmDN BRG1 |
| GGE_208 | DH5α | pV2 (GGE140) EcoRI-NotI | ArmUP Gzf1 – Nat – ArmDN Gzf1 |
| GGE_209 | DH5α | pV2 (GGE140) EcoRI-NotI | ArmUP Hsf1 – Nat – ArmDN Hsf1 |
| GGE_211 | DH5α | pV2 (GGE140) EcoRI-NotI | ArmUP Hac1 – Nat – ArmDN Hac1 |
| GGE_212 | DH5α | pV2 (GGE140) EcoRI-NotI | ArmUP Klf1 – Nat – ArmDN Klf1 |

**Table S2**. List of *Y. lipolytica* strains used in this study. Po1-series strains are: Po1f, ATCC MYA-2613, genotype*: MatA, leu2-270, ura3-302, xpr2-322, axp-2*; and Po1h: *MatA, leu2-270, ura3-302, xpr2-322, leu2‑270, axp-2*. All the following strains are derivatives of the Po1 series.

* reference (Leplat et al., 2018, 2015); JMY2566: *MATa, ura3::pTEF-RedStar2-LEU2ex-Zeta, leu2-270, xpr2-322; JMY2810: MATa, ura3::pTEF-RedStar2-LEU2ex-Zeta-URA3ex-pTEF, leu2-270, xpr2-322*

** reference (Onésime et al., 2025)

If not marked, a strain was constructed in this study.

| ECY number | Parental strain | modification | Phenotype |
| --- | --- | --- | --- |
| ECY_220 | Po1h | JMP62-scYFP | scYFP |
| ECY_181 | JMY2566* | pTEF-Dep1/Azf1-OE at the specific URA3-zeta | OE Dep1, OE inRFP* |
| ECY_265 |  |  | OE Azf1, OE inRFP* |
| ECY_177 | JMY2810* | KO Dep1 Dep1-ArmUP-NATr-Dep1-ArmDN | KO Dep1, OE inRFP |
| ECY_282 |  | KO Azf1 Azf1-ArmUP-NATr-Azf1-ArmDN | KO Azf1, OE inRFP |
| ECY_307 | Po1f leu2-ura- | pJMP62-ura3-scYFP + pJMP62-leu2-TF37 | scYFP-TF37 |
| ECY_313 | Po1f leu2-ura- | pJMP62-ura3-scYFP + pJMP62-leu2-KLF1 | scYFP-KLF1 |
| ECY_321 | Po1f leu2-ura- | pJMP62-ura3-scYFP + pJMP62-leu2-HSf1 | scYFP-HSF1 |
| ECY_352 | Po1f leu2-ura- | pJMP62-ura3-empty + pJMP62-leu2-empty | ura+ leu+ (control FL-) |
| ECY_355 | Po1f leu2-ura- | pJMP62-ura3-scYFP + pJMP62-leu2-HAC1_intronless | scYFP-HAC1 |
| ECY_417 | ECY599 | pJMP62-ura3-scTlG + pJMP62-leu2-SFP1_intronless | scTlG-SFP1 |
| ECY_420 | ECY639 | pJMP62-ura3-scYFP + pJMP62-leu2-Hsf1_intronless | scYFP-Hsf1 |
| ECY_423 | ECY639 | pJMP62-ura3-scYFP + pJMP62-leu2-Gzf1_intronless | scYFP-Gzf1 |
| ECY_426 | ECY639 | pJMP62-ura3-scYFP + pJMP62-leu2-Klf1_intronless | scYFP-Klf1 |
| ECY_572 | ECY599 | GGA-ura3-pTEF-SP3-scTlG + pJMP62-Leu2-HAC1 intronless | scTlg-HAC1 |
| ECY_669 | ECY599/600 sp3_pTEF_TlG leu2- | JMP62 leu2 empty | ura+scTlG leu+ |
| ECY_672 | ECY599/600 sp3_pTEF_TlG leu2- | JMP62 leu2-Gzf1 | ura+scTlG Gzf leu+ |
| ECY_732 | ECY639 (Po1f leu-ura- + JMP62 ura3-scYFP | pJMP62-ura3-scYFP + pJMP62-leu2-Hap1 | scYFP_Ura3_Hap1_intronless_Leu2 |
| ECY_735 |  | pJMP62-ura3-scYFP + pJMP62-leu2-Mhy1 | scYFP_Ura3_Mhy1_Leu2 |
| ECY_738 |  | pJMP62-ura3-scYFP + pJMP62-leu2-Yas1 | scYFP_Ura3_Yas1_Leu2 |
| ECY_741 | ECY599 (Po1f leu-ura-+ JMP62 ura3-SP3-pTEF_TlG ) | pJMP62-ura3-scTlG + pJMP62-leu2-Klf1 | scTlG_ura3_Klf1_intronless_Leu2 |
| ECY_744 |  | pJMP62-ura3-scTlG + pJMP62-leu2-Hsf1 | scTlG_ura3_Hsf1_intronless_Leu2 |
| ECY_747 |  | pJMP62-ura3-scTlG + pJMP62-leu2-Hap1 | scTlG_ura3_Hap1_intronless_Leu2 |
| ECY_752 |  | pJMP62-ura3-scTlG + pJMP62-leu2-Mhy1 | scTlG_ura3_Mhy1_intronless_Leu2 |
| ECY_755 |  | pJMP62-ura3-scTlG + pJMP62-leu2-Yas1 | scTlG_ura3_Yas1_Leu2 |
| ECY_782 | ECY639 (Po1f leu-ura- + JMP62 ura3-scYFP | pJMP62-ura3-scYFP + pJMP62-leu2-Sfp1 | scYFP_ura3_Sfp1_intronless_Leu2 |
| ECY_789 |  | pJMP62-ura3-scYFP + pJMP62-leu2-Msn4 | scYFP_ura3_Msn4_Leu2 |
| ECY_792 |  | pJMP62-ura3-scTlG + pJMP62-leu2-Msn4 | scTlG_ura3_Msn4_Leu2 |
| ECY_795 | ECY758 | pJMP62-ura3-scTlG + pJMP62-leu2-empty + pJMP62-lys5-empty | scTlG_ura3_empty_Leu2_Lys5_solo |
| ECY_798 | ECY762 | pJMP62-ura3-scTlG + pJMP62-leu2-Hsf1 + pJMP62-lys5-Gzf1 | scTlG_ura3_Hsf1_Leu2_Gzf1_Lys5 |
| ECY_802 | ECY762 | pJMP62-ura3-scTlG + pJMP62-leu2-Hsf1 + pJMP62-lys5-Klf1 | scTlG_ura3_Hsf1_Leu2_Klf1_Lys5 |
| ECY_805 | ECY764 | pJMP62-ura3-scTlG + pJMP62-leu2-Gzff1 + pJMP62-lys5-Klf1 | scTlG_ura3_Gzf1_Leu2_Klf1_Lys5 |
| ECY_831 | ECY773 | pJMP62-ura3-scYFP + pJMP62-leu2 empty + Lys5 solo | scYFP_Ura3, Leu2+, Lys5+ |
| ECY_913 | ECY_351 (eyk1Δ Ura3- Lys5- Leu2- Fil-, JMY8695** / triple auxotroph)) | pJMP62-ura3 + pJMP62-leu2 empty + Lys5 solo | Ura3+, Leu2+, Lys5+ |
| ECY_985 | ECY220 (Po1f (scYFP_Ura3_emptyLEU2) | KO Dep1 Dep1-ArmUP-NATr-Dep1-ArmDN | K.O. Dep1 scYFP |
| ECY_988 |  | KO Euf1 Euf1-ArmUP-NATr-Euf1-ArmDN | K.O. Euf1 scYFP |
| ECY_989 |  | KO Azf1 Azf1-ArmUP-NATr-Azf1-ArmDN | K.O. Azf1 scYFP |
| ECY_993 |  | KO Gzf1 Gzf1-ArmUP-NATr-Gzf1-ArmDN | K.O. Gzf1 scYFP |
| ECY_1003 |  | KO Yas1 Yas1-ArmUP-NATr-Yas1-ArmDN | K.O. Yas1 scYFP |
| ECY_1008 |  | KO Msn4 Msn4-ArmUP-NATr-Msn4-ArmDN | K.O. Msn4 scYFP |
| ECY_1009 |  | KO Hsf1 Hsf1-ArmUP-NATr-Hsf1-ArmDN | K.O. Hsf1 scYFP |
| ECY_1012 |  | KO Hac1 Hac1-ArmUP-NATr-Hac1-ArmDN | K.O. Hac1 scYFP |
| ECY_1015 | ECY423 (Po1f (scYFP_Ura3_Gzf1intronless_leu2) | KO Dep1 Dep1-ArmUP-NATr-Dep1-ArmDN + OE Gzf1_in Leu2 | K.O. Dep1 scYFP - Gzf1 OE |
| ECY_1019 | ECY834 (ECY_351 (eyk1Δ Ura3- Lys5- Leu2- Fil-, JMY8695 / triple auxotroph)**) | pJMP62-ura3-scYFP + pJMP62-leu2 Gzf1 + pJMP62-lys5-Klf1 | scYFP_Ura3_Gzf1_leu2_Klf1_lys5 |
| ECY_1022 |  | pJMP62-ura3-scYFP + pJMP62-leu2 Hsf1 + pJMP62-lys5-Klf1 | scYFP_Ura3_Hsf1_leu2_Klf1_lys5 |
| ECY_1025 |  | pJMP62-ura3-scYFP + pJMP62-leu2 Hsf1 + pJMP62-lys5-Gzf1 | scYFP_Ura3_Hsf1_leu2_Gzf1_lys5 |
| ECY_1028 | ECY220 (Po1f (scYFP_Ura3_emptyLEU2) | KO Cat8 Cat8-ArmUP-NATr-Cat8-ArmDN | K.O. Cat8 scYFP |
| ECY_1030 | ECY426 (Po1f (scYFP_Ura3_Klf1_Leu2) | KO Dep1 Dep1-ArmUP-NATr-Dep1-ArmDN | K.O. Dep1 scYFP - Klf OE |
| ECY_1031 | ECY220 (Po1f (scYFP_Ura3_emptyLEU2) | KO Sfp1 Sfp1-ArmUP-NATr-Sfp1-ArmDN | K.O. Sfp1 scYFP |
| ECY_1033 |  | KO Hap1 Hap1-ArmUP-NATr-Hap1-ArmDN | K.O. Hap1 scYFP |
| ECY_1036 |  | KO Mhy1 Mhy1-ArmUP-NATr-Mhy1-ArmDN | K.O. Mhy1 scYFP |
| ECY_1046 |  | KO Klf1 Klf1-ArmUP-NATr-Klf1-ArmDN | K.O. Klf1 scYFP |
| ECY_1052 |  | KO TF024 TF024-ArmUP-NATr-TF024-ArmDN | K.O. TFo24 scYFP |
| ECY_1055 |  | KO Gzf1 Gzf1-ArmUP-NATr-Gzf1-ArmDN | K.O. Brg1 scYFP |

**Table S3**. A simplified list of *Yarrowia lipolytica* strains used in this study. “X” denotes a strain constructed and studied for phenotype. OE – overexpression, KO – knock-out, scYFP – secretory form of YFP, and scTlG – secretory form of the glucoamylase (derivatives of Po1f). Double mutants (two TFs modified by OE or KO) are derivatives of JMY8695.

| target/modification | scYFP | | scTlG |
| --- | --- | --- | --- |
| TF | OE | KO | OE |
| Hac1 | X | X | X |
| TF024 |  | X |  |
| Gzf1 | X | X | X |
| Euf1 |  | X |  |
| Hsf1 | X | X | X |
| Yas1 | X | X | X |
| Mhy1 | X | X | X |
| Msn4 | X | X | X |
| Hap1 | X | X | X |
| Cat8 |  | X |  |
| Sfp1 | X | X | X |
| Azf1 |  | X |  |
| Klf1 | X | X | X |
| Dep1 |  | X |  |
| Brg1 |  | X |  |
| Klf1-Hsf1 | X |  | X |
| Klf1-Gzf1 | X |  | X |
| Gzf1-Hsf1 | X |  | X |
| Klf1-ΔDep1 | X | |  |
| Gzf1-ΔDep1 | X | |  |

**Table S4**.

Codon-optimized, synthetic versions of TFs for OE without *BamHI* and *AvrII* restriction sites inside the sequence, without introns, with *BamHI* and *AvrII* overhangs. HAC1 - intron 30 bp removed, SFP1 - intron 803 bp removed, KLF1 - two introns removed, HSF1 - R68S mutation for continuous activation introduced (homologous to R206S). Illustration in Figure S1.

| Gene | Sequence |
| --- | --- |
| HAP1 | AAGGATCCATGACCGACCAAGAGTCTCGACGAAACGGCCTCGAGCAGCTGGCCGTGCGAGCCATTGCCGAGATGCGAGAAGGCCAGGGACCTCGACCTCCTGACACTCGAGATCAGCAGCCCCTGGACCTGCTGCCTGACCACGAGGACAAGTCTGTGAACAACAACGGCACCCGACTGGCTCCCTACGAGCAGTGCATCCCCGACCTGTCTGCCCCTGGCGCCGGATTCGAGGACGACTTCTCTCGAGTGGTGAAGCGACGACGACGAGCCCCTGTGTCTTGCCTGCTGTGCCGAAAGCGAAAGGTCCGATGCGACAAGCAGATGCCCTGCTCTGCCTGCAAGACCGCCAACGTGACCGGCTCTTGCGAGTACGCCCCTCCTACCTGGGGCGGACGAGAGGTGCGAGGCGGCCAAGAGATCCAGTTCACCGTGGACGAGGCTGAGCCCATCTCTCAGCCTCCAGGCAACAACTCTTCTAACTCTGACACCCGAGGCCACCACTCCTCTTCGTCCTCTTGCTCTAACATCTCTGTGCCTCCAATGCAGAAGCCCCTGACACCTCCTCAGATGCTGCAGCCTATCGTGTCTCCTCTGCAGCCCACCGTGAAGTCTAAGTCCTCTCACCAGCGAATGTACGGACGATTCCCCAACGCCGCCAACCTGCTGCGAGCTGACCTGTCCGAGCGACAGCGAGCCAAGGGCGTCGAGGTGCCTCCTTCGCCTCGAAAGAAGCAGCCTCTGCACAACGACGCCAAGTGCAAGCGAGCTATTGAGACTCTGCAAGAAACCCGACGACGAAGATCTGACTCTCCTATCGACCCCGTGTACAAGCCCCTGCTGGATCTGTTCCCCGGCCAGCAGTACTGTGAGTTCCTGATCGACTCGTTCCTGAAGCGAGTGAACACCGTGCACTACTGCGCTTGCCCCGAGGGCCTGCGAACCGACTTCAACGCCCTGTGGTCTGCCAAGCAGCGAATCGAGGACGGCGAGCAGTTCAACGCTCGAACCTACCTGAACCTGCCTTCTATCGCCGTGATGCTGCTGGTGTTCCGACTCGGACGAGGATCTTACCCTCTGAAGGACTGGTCCCCTCCTAACTACTGCATCAAGGACGGCCACCGAGACAACACCTACCTCGGACCTCAGGTGCTCGAGGTGGCCGAGGCCTGCCTGAACGCCTCTCACATCTTCCGACACGGCGGCCTCAAGACCATTCAGGCCCTGGTGCTGATGAAGCTGGACTGCCTGTACGCTCCCGACTCTGAGTACTCTCCCGACGGCGTGGACTCTGTGAACCTGACCGGCGTGATCCTGCAGCTGGCTCTGTCTCAGGGCCTGTACATGGACCCCGCCAACTTCGACACCCAGTCTACCTCTGTGCTGGCCTTCCAGGACTCTCTGCTGGTGCAGTCTCCCAAGGCTCACCGACGATCTATGTCTGTGTCCTCTGCCGGCTCTGCCTCTCTGATCGAGCCCTCTTCGGCCAAGCTGTGGCGAATCCTGTACGGCTGCGCCCTGATCCTGGACGCCAAGCGAAACCTGGAAGTGGGCATTCCCCTGTCTCTGCCCCAGGGCGACTGCGACGTGTACTTCGACCACACCGACGACGGCGGAAAGACCGCCTCTAACGTGCTGGCTTTCCGACGAGCCATGCTGCGATTCTGTGCCCTGTCTGGCTGCGTGATGACCGAGACTTCTCGACCCGTGATGTACCGAAACGAGCCTCTGATCCAGCGACTCGGCCAGGACCTGCACAAGTTCGGCGTCGACGAGGTCGCCTCTCTGGCCCAGCTGCGAGAGGAACTGGGAATCCCCAAGGACGACTCCCTGGCTTTTTCTTCTGACGACGACCACGCTGACGAGTCCGACACCTCTTCTGTGGCCTCTTCTGCCACCTCTGCTCTGCAGCTGATCCACCGATGCCGAATCTACTGCCTGTGGTCCAAGCTCTGTGTGCACTACGAGATGGACACCTACGACGCCTCTGAGGAACCCCTGTACGGACACCGAGAGCTGTGCGTGCGACTGGGCATCGAGCAGGACCAGGCCGGCGTGCTGTCTCAGTACCGAGTGATTCTGCGAATGTCCTCGCTGATCACCCTGCTCGTGTGGGTCGCCCGACGAAGAACCGCCTCTGTCGAGCTGGGAGCCCACTCTTGGTACATCGTGTCCCTGGTGCACTCTATGATGGGACTGCCCCTGACCGGTGTCATCACCTGTTGGTTCCGAGTGGCCATCCTGGCCAAGGAAACCGGCTCTCTGAAGGGCACCCGATGGGACCCTGACTGGCTGTTTCGACTGATTACCCAGGGAATCGCCGTGCTGGGCGACACCGACGTGGGCGACCGACAGATGCACGGCCTGTGCGTGTCTTTCCTGAAGAAGGCCCGATCTGACCTGGGCCGACTGCTGGCTAAGATGCCCGGTTCTGACCCCGCCTACCTGTCCTCTCAGTTCGACGCCATCGACAAGCTGGCCGAGGTTGAGGTGCTGTCTGAGGAAGATGAGGGACTGTAACCTAGGTT |
| HAC1 | aAGGATCCATGTCCATCAAGCGAGAGGAATCTTTTACCCCTACTCCTGAGGACCTGGGCTCTCCCCTGACCGCCGACTCTCCCGGCTCTCCCGAGTCTGGCGACAAGCGAAAGAAGGACCTGACTCTGCCCCTGCCTGCCGGCGCTCTGCCTCCTCGAAAGCGAGCCAAGACCGAGAACGAGAAGGAACAGCGACGAATCGAGCGAATCATGCGAAACCGACAGGCCGCTCACGCTTCTCGAGAGAAGAAGCGACGACACCTCGAGGACCTTGAGAAGAAGTGCTCTGAGCTGTCCTCTGAGAACAACGACCTGCACCACCAGGTGACCGAGTCTAAGAAGACCAACATGCACCTGATGGAACAGCACTACTCTCTGGTGGCCAAGCTGCAGCAGCTCTCTTCTCTGGTGAACATGGCCAAGTCCTCTGGCGCCCTGGCCGGCGTGGACGTGCCCGACATGTCTGACGTGTCTATGGCTCCCAAGCTCGAGATGCCCACCGCTGCTCCCTCTCAGCCTATGGGCCTCGCCTCTGCTCCCACTCTGTTCAACCACGACAACGAGACTGTGGTGCCTGACTCTCCCATCGTCAAGACCGAAGAGGTGGACTCTACCAACTTCCTGCTGCACACCGAGTCCTCTTCGCCTCCAGAGCTGGCCGAGTCTACCGGATCTGGATCTCCCTCTTCTACCCTGTCTTGCGACGAGACTGACTACCTGGTGGACCGAGCCAGACATCCCCAGCGACGGCACAAGATTTCTTTCTCTTCTCGAACCTCTCCTCTGACCACCTCTCTGGACTGCATGGACTGCCGAATGACCTCGCCTTGCCTCAAGACCACCTCCTCGCTGCCCTCTACCACTCTGCTGCTGATCTAACCTAGGTT |
| SFP1 | AAGGATCCATGACCGCTACTCCCATCGACATCTCTTACCAGCAGAACTTCCACTCTCAGCGATTCCGACGAGAGTCTATGGCCCACTCTCAAGGCATGGGCGGCATCTCTTGGGGCGGCATGTCTGTCGGCTCTTGGCTGCGAGATGAAGAGGAATCTCCCGAGGTGCCCTCTACCTCTCCTTCTAACACCAACCACCGAGTGATGACCCTGAACATGCCCTCCACCACCACCATGGCTATGTCTCCCGGAATGGGAATCAACTCTTCTTCTTACCTGCCTAACCTCGAGGCCCAGTACTGCAAGGACTACTCTTGCTGCGGACAGACCCTGCCTACTCTGCACGACCTGCTGCGACACTACGAAGAGTCTCACATTGCTCCCTCGCCTCCAGTCGAGCAGCCCCACATGGCCCGAAACACCAACAACGTGATGGAAGTGGTGTCTACCAACGACGTGTTCATGTCTATGGGAGGACCCGGCGGACCTCCTGGCCAGCAGGGCGCCCAGGGCATGCAGCATCACCCCATGGCTCAGGGCCACCATCCTACTCACTTCCAGCAGCACCCTCTGCCTCCACAGGGACACGCTCCCCAGGGCACCCAGATGCCTCACGGCCAGCACAACCCTCAGATGATGCAGATGGGCCAGCCTCCTCAGCCTACCGGCACCTTCGACGAGGACGACGAGATGTGCATCGACGACCCCGCTCGACACCTGTACGTCATGGAACGAGGCGAGCACAAGCCCTTCAAGTGCCCCGTGATCGGCTGCGACAAGACCTACAAGAACCAGAACGGCCTGAAGTACCACCGAATGCACGGCCACCAGAACCAGATGCTGCACCTGAACGACGACGGCACCTACTCTATCATCGACCCTCTGTCTAACGCTCCCTATCCTAACGGCGTGGACATGGAAAAGGACAAGCCCTACCGATGCGAGATCTGCGGCAAGCGGTATAAGAACCTCAACGGACTCAAGTATCATCGAGGCCACTCTACCCACTAACCTAGGTT |
| KLF1 | AAGGATCCATGTCCGACGACCGGAAATTCAAATGCAAGCACCCGGGGTGCGAAAAGACCTTCAACAGACGGGATTATCTCATACGACACAGTGCTAACCACCTCGACACGCTGCCTTTTCAATGTCCGCAGTGTCCCATGAAGTTTGCCCGAGCAGACTTGCTGCAAAAGCACCTCAACACGCGCTCGCACGAACGACGAAAGAAGCGAGAGGAGAAAGAAAAAGCGAAAATCGAGCGTAAACAACGACGCAACTCCATGAAGTCCCAGAGCAGGAGCAACTCGATCGACATGAACACCAACATTGTGTCGCCCACCACCGACAATTACACATGGCTGTTTGGCGCCCAGTGGCCCGAGTCCGCCACCACAGCCGAAGACACGTTCAAAATCAAGAGCGAGTTTGTCCCACATCAGGAGCCGACGCAGCCCGACAAGAACACCAGCGAGCTGGACTTCATGCACATGGTGGAGTTTGAACAGCAGGGCCAGGGTCGTGCCTTTTATGCCGGCATCAAGACCGACCTCGACAACTCAGACACAACCAGTGACTGCTCTATCATGTCACAGTACTCCAACTCCACCCAGGGCACCTATCCTCGCTCCTCGGTCAACATGCCCGACCTGACGCTGCCTCCCCATATGCGAATGTCTGTGGCCTCCGTCACTTCAGCTCCCTCCATGCCCCACGTGCCACACATGCTCCAGCATCAACCCCAGCACCGCGCGGAGCTGCAACCGCCACACACACATCTGATTCAGCAGCAACCGCCGCCACCACCCCAGCATGTCCATGGCGTTTCTCCAGAGATACTTAATCCATCACTGGGAATCCAGCCTGTGTGTCTCAACACCATCGGCGACCTGACACCTCCCGCCGAGCCTTTTGTGGCCCGAGAAGACAAACTGGCCCAACAGTCAGCAAATGCAAACCAGCAACATCTCCCCACGCCGCAGACCCACACACAGAGCCAGACACAACCGACGCAGCCTTTGTCGCCCCGTGACAAGTACCTGGGGCTTTTCTGGAGCAACTTTGACCAGCTGTACCCCATCATCCACAGACCGTCATTCAACCCGGACACGGCTCCCGAGGCGCTCGTGACTACCATGGTGTGTGTCGGAATGACCTACGACTCCGAGCCTGCAGTCTACCAGGAGGCTATCCGCACCATGCAGCAGCACGTGCATCAGCTGTACTCCAAGGCTGCTTTGGTACGGGGCACCAATGCAGATGAAAACTTGTGGGTGCTTCAGGCCTTGGCCCTGGCTGCCTACTTCCACCGGCTTCTGGGCTCCAAGGACCAGTGGGAGCTCGCCGAAATGTACTCTTGCATTGCTCTGGTTAAGGACCAGATGGTGACCGAGAACGCTCCTCAGCTGGGCCCCGACATTGCTGCGTTTTGGCAGGTGTGGATCAAGCACGAGGCTCGAAAACGGCTGCTGTGCTTCTTCTTCATCCTTGACACCCAAGCCGCAATTCTCTTCAAACACACCTCTCACCTGTCTGTGTTTGATACGGATCTGGAGCTTCCCTGCACTGATGCTGCCTGGAGTGCCAGAGACACTCGGGAGTTTTACATTGTGTACACTCTCCAGCCCCGAGGTCCTAACAACCTGCGACCTGCTCCTGACGTGACCGAGACGCAGTCTTACCCCAAGAACAAGCCGTCGTCTGCTTCTCGGCGATTCGACAACGAGTGTCATCGTGACTGGCCCTCTCTCCTGTTTTGTGTCAAGCGAATCATGAGTGACTACGAGGAGGACCAGCAGGAGTACCCTCTGGCCTGTTTCTCGCAATTTTCGCGTCTGCTGCTGCTCCATGGCATCCTCAACATCTGTCTGGATCTCCAGCACCGAATGGTGTCAAAGAAGACGGATTACAAGACCAAAATGGTTGTTGCGTTTATGTCTTGGAGAGGCTACTTTGACCGGCAGCTTCGGGAAATCAACCGGGAATTCCAGTTGACCAACATCATGGCTGAGAACGCAGCTCATCTCAAGGACGAGACCAACGCCAGTGCTCTTCTTGGCAACATGTCTGGCAATGAGGACATTACTCTGCAGTTCAACAACTACGGTAACACTCCTCTGCTGTGTTCCAACTGGTCGCTGTTCTCCGTGGGTCTTATGACTCTGTTTGTGGACACCCAGATGCTTCGCCAGTACACTGGCACCGTTTCTTCTCCCTCCGAACGGGTCACTGCCACTCTCCGGCAGTGGGCCAACGGCCATGACGTGACTTGGGCTGTCTGGTACGCCAACCAGCTTCTTGGCCGGGTGTTCCAGAACGAGGCTCTGCCGTTTTTGGCGGACCACGTGGCCTGGGGCATGTTTTCCGCCTGTCTGACGCTCTACACTTTTGAGTCGCTACGACGGTCCAACTCGCCCCTCCAGTCGCCCCTGGAGTCGATTTCCGAGTCTCCCCGGGTTGAGAACACCCAGAGCGAGGCTCTGGAGTACATTCGGCTGGCCTCGCAGACTCCTCCCGAAGCCATTGACGACTTTATTTCCGAGTCGGCTGCCACGACTCTCATGCGCCAGAAATACATTGTTTCTGTGCTTCGTCACACCGCCAATCTTCTGTCTTCATCGAGCAGAGAGCAGTGTGTCTACATTGTCTCACATCTTAATGGCATTCTTGCCCGTGTATAACCTAGGTT |
| GZF1 | AAGGATCCATGAACCAGAACCTGACCGTGGCCATCAACCAGGGCCACCTGTCTAACCAGCGATCTCAGTCTGTGGTGGACATGTCTCACATCTCTACCGCTCGAAAGCAGTCTAAGTCTGGCACCTCTACTCCCCGAGGACTGCCCCGAACCATGTCTACCTCTAACATTCCCCAGCAGCGAATCTCTAAGCCCAAGACCAACGTGTCCTCTACCAACGTGAAGGCTGGCGCCAACACCGTGACCGCCGACGACAAGCCCACCTCTTGCACCAACTGTCACACCCAGACCACTCCTCTGTGGCGACGAAACCCCGAGGGCGAGCCCCTGTGCAACGCCTGCGGCCTGTTCCTGAAGCTGCACGGCGTGGTGCGACCCCTGTCTCTCAAGACCGACGTGATCAAGAAGCGAAACCGAACTAACGGAACAAACGCCTCTTCTTCTGGCACCGGCGGAGCCTCCACCAACACTCCCTCGCCTGACTCTGCCAAGGAAACCGCCTCTACCGCCACTCGAAAGGGCGGCTCTCGATCTCGATCCCGAAAGAAGGAAGAGGCCACCGTCAAGGGCGACACCGCTCAGTCTACCCCTACTCAGCCCTCTACTCAAGAGTCTCCCGTGGGCATCGTGCCCAACAAGACTGACTCTGAGGAACTGAAGATGTTCGGAGCCGCCAACGTCGAGGAACGAGATGGCCAGTGGGAGTGGCTGTCTATGGCCCTGTAACCTAGGTT |
| HSF1 | AAGGATCCATGTCTAACACCCTGGTGAAGAAGGGCCAGGGCCAAGCCGCCTCTAACGCCAAGAAGATCGGCTACAACAAGAAGAAGGGCGGAGGCCAGACCAAGGGACGACCCGCCTTCGTGTTCAAGCTGTGGAACATGGTGAACGACCCCGCCTACGACGAGTACATCCGATGGATGCCCGACGGCAAGTCTTTCCAGATCACCGGCTCTGAGCAGCTCGAGAAGATCGTGCTGCCCCGATTCTTCAAGCACAACAACTTCTCTTCGTTCGTGCGACAGCTGAACATGTACGGCTGGCACAAGGTGCAGGACGTGACCTCTGGCTCTATGCAGTCTAACGACGAGGTGTGGCAGTTCAAGTCTCCCAACTTCATCCGAGGCCGAGAGGACCTGCTGGACAACATCGTGCGAAACAAGGGCACCAAGGGCTCTGACGAAGAGGAAGAGATGGACATGACCACTCTGATGGACGAGCTGCAGCAGATCAAGCTGAACCAGCTCAACCTGACTCAAGAGGTGAACAAGCTGCGAACCGACAACCAGCTGCTGTGGCAAGAGAACCTGGGCTTCAAGGACAAGCACAAGCAGCACGGCGAGACTCTCGAGCGAATTATGCGATTCCTGGCCTCTCTGTACGGCAACCAGGGCAAGGTGCTCGGCGAGGCCATCTCTGGCAACCGAAACCAGAGACTGCTGCTGGGCAACACTGACCCCGCTCAGGCTACCCACATGATCAACGAGGCCGCTGCCGCCGCTGCTGCCGAGAACTCTGGAAACTTCCCCTTCGAAATGAACGACATGTTTAACAACAACAACAACAACAACAACAACTCGAACAACCCCAACGACTCGCCCTCGTTCGCCCGAGTGCAGCCCCTGTCCTCTACCAACTCTCCCCAGACCGTGACCGGCTCTCCCCGAGAGGAAAACAACAAGACTAACAACACAAACAACACGTCCAACAACTACGACAAGATCACCGCTTACGACACCGACACCGGTTCCGAGCTGCCCGACAACCTGCAGGACCTCACCGACCTGTCTGACTTCACCCCTTCTATGCTGGACCCTCAGACCGTGCAGCAGTCGATGGAACCTCCTATGGGCTTCGGCGACCTGAACAAGAACTCTTCTCTGATCCTGTCAAACAACAGTAACAACTCTATCAACATGCCCCAGACTCCTATGAGAAACAACTCCACCTCTTCGCACTCTTCTCAGAACATGTCTCACCACAACTACAACTACCAGCCTTCTACTCCCGGCGCTCTGTTCCCCGAGCTTGAGGCCTTCCAGGGCGCCAACGACCAGCTGATCCGATCTGCCGAGCGACCCCGAAACAACCTGAACATCAACGGCAACGGCAAGGCTGACGACCTGCAGCGACAGCTCCAGGACCACGGCCGACACATCGACCAGTTCACCGACCGACTGAAGAAGCAGCAGGGATACCAGCCTCAGCAGCTCCAGCATCAGCAACAGCCCCAGCAGCCTCAACAGCAGCCCACTCCTATGCAGGCTCCCGCTCCTACTCCTGCTTCTACCATCTCCTCTGCTCCCACTCCTGATCCTACCTCTGACCTGTTCAACGTGGGCGACCCCAACATTGTGCCCATGCAGGACTTCAACATGGACGACTACATCGACAACGAGGCTGACATGTTCGACCCCGAGGTGAAGATCGAAGAGGACATGCCCCTGGTGACCGAGCTGGACTCTCCTGCTCCTCGACCTAAGAAGCGAAAGGCCGGCGAGTAACCTAGGTT |
| MHY1 | AAGGATCCATGGACCTGGAACTCGAGATCCCCGTGCTGCACTCTATGGACTCTCACCACCAGGTGGTCGACTCCCACCGACTGGCCCAGCAGCAGTTCCAGTACCAGCAGATCCACATGCTCCAGCAGACCCTGTCTCAGCAGTACCCTCACACTCCCTCTACCACTCCTCCAATCTACATGCTGTCTCCCGCCGACTACGAGAAGGACGCCGTGTCTATCTCTCCCGTGATGCTGTGGCCTCCTTCGGCTCACTCTCAGGCCTCTTACCACTACGAGATGCCCTCTGTGATCTCTCCCTCTCCTTCTCCTACTCGATCTTTCTGCAACCCTCGAGAGCTGGAAGTGCAGGACGAGCTGGAACAGCTCGAGCAGCAGCCCGCTGCTCTGTCTGTCGAGCACCTGTTCGACATCGAGAACTCTTCTATCGAGTACGCCCACGACGAGCTGCACGACACCTCTTCGTGCTCTGACTCTCAGTCGTCTTTCTCGCCCCAGCAGTCTCCCGCTTCTCCCGCCTCTACCTACTCGCCCCTCGAGGACGAGTTCCTGAACCTGGCCGGCTCTGAGCTGAAGTCTGAGCCCTCTGCTGACGACGAAAAGGACGACGTGGACACCGAGCTGCCTCAGCAGCCTGAGATCATCATCCCCGTGTCTTGCCGAGGACGAAAGCCCTCTATCGACGACTCTAAGAAGACCTTCGTCTGCACCCACTGCCAGCGACGATTCCGACGACAAGAGCACCTGAAGCGACACTTCCGATCTCTGCACACCCGAGAGAAGCCCTTCAACTGCGACACCTGTGGCAAGAAGTTCTCTCGATCTGACAACCTGGCTCAGCACATGCGAACCCATCCTCGAGACTAACCTAGGTT |

**Table S5**. Oligonucleotides used in this study

| Primers for TF amplification from genomic DNA for OE, with *BamHI* and *AvrII* overhangs. | |
| --- | --- |
| Primer | Sequence 5’ à 3’ |
| Msn4_BamHI_F | aggatccATGACCACCTACGATCTCAAC |
| Msn4_AvrII_R | acctaggTTAACATCGCCGCTTCTGTCG |
| Yas1_BamHI_F | aggatccATGGATTCCCGATCAGCCTCC |
| Yas1_AvrII_R | acctaggCTAGACCGGAGACTCCTCTTTC |
| Primers for TFs’ homologous arms amplification from genomic DNA for KO. | |
| Primer | Sequence 5’ à 3’ |
| ArmUp_Msn4_EcoRI_F | agaattcTGTATTAGGGTTGGGGTTAGGG |
| ArmUp_Msn4_KpnI_R | aggtaccGCCCTTGACAAACGACTGGGGC |
| ArmDn_Msn4_BamHI_F | aggatccTGCGACAAGCGGTTCAAGCGCC |
| ArmDn_Msn4_NotI_R | agcggccgcTGGCTGGAGTTGACTATCAGCG |
| ArmUP_Mhy1_EcoRI_F | aGAATTCGTGGTGTTGAAAAGGTGAAAAAGAG |
| ArmUp_Mhy1_KpnI_R | aGGTACCGGATTACAGAAGGATCTAGTGGGAG |
| ArmDn_Mhy1_BamHI_F | aGGATCCGCAGCCCGAGATCATCATCCC |
| ArmDn_Mhy1_NotI_R | aGCGGCCGCACACACTGCCGGGGGTATTTCT |
| ArmUp_Yas1_EcoRI_F | aGAATTCAGCACAGCCATGAAGCACTCTG |
| ArmUp_Yas1_KpnI_R | aGGTACCTGCTCGCTGGCAATGTGGTTGG |
| ArmDn_Yas1_BamHI_F | aGGATCCGCGGAGGGCAAGGAGCTCGAGC |
| ArmDn_Yas1_NotI_R | aGCGGCCGCCGCCGCAGCTACAGTTGGCGCC |
| ArmUp_Hac1_EcoRI_F | aaGAATTCCGCTATAACCATGGCCAGTGGA |
| ArmUp_Hac1_KpnI_R | aaGGTACCCTTGGCCATGTTGACGAGCGAC |
| ArmDn_Hac1_ PfoI_R | TCATTTCATTCCAGGACACAAGTGTCTT |
| ArmDn_Hac1_NotI_F | aaGCGGCCGCTGGGTCTCGCCAGCGCGCCCAC |
| ArmUp_Sfp1_EcoRI_F | aGAATTCGGCCTTGACCAACCAAACCCCG |
| ArmUp_Sfp1_KpnI_R | aGGTACCAAGAGGCTCTGCGGTAACAAGC |
| ArmDn_Sfp1_BamHI_F | aggatccCTCGAGGCGCAGTACTGCAAGG |
| ArmDn_Sfp1_NotI_R | aGCGGCCGCCGAACCAGAACTCGTAATGTAG |
| ArmUp_Klf1_EcoRI_F | aaGAATTCCATACGAGGCGGATCTCAAGAC |
| ArmUp_Klf1_KpnI_R | CAGGTACCTTTCGGCTGGGGTGGTATTTTTG |
| ArmDn_Klf1_NotI_F | aaGCGGCCGCAGTTGACCAACATCATGGCTGA |
| ArmDn_Klf1_PfoI_R | aaTCCaGGAGCTGCTCTCTATCTTTGCAATG |
| ArmUp_Hsf1_EcoRI_F | aaGAATTCTCTTGCAGTAGCCCGATGAAGA |
| ArmUp_Hsf1_KpnI_R | aaGGTACCTTGTGCCACCCGTACATGTTGA |
| ArmDn_Hsf1_PfoI_R | aaTCCaGGAGATGGCAAGGATAAAAAGTGTG |
| ArmDn_Hsf1_NotI_F | aaGCGGCCGCGATACTCTCTAACAACTCCAAC |
| ArmUp_Hap1_EcoRI_F | aaGAATTCCGGCCTGCAAGACTGCTAAC |
| ArmUp_Hap1_KpnI_R | aaGGTACCATGTACAGACCCTGCGAGAG |
| ArmDn_Hap1_BamHI_F | aaGGATCCGTTGTCGTTACCTCAGGGCG |
| ArmDn_Hap1_NotI_R | aaGCGGCCGCGGCATCTTGGCCAGCAAACG |
| ArmUp_Gzf1_EcoRI_F | aaGAATTCCATGATAATGATGATAACGATG |
| ArmUp_Gzf1_KpnI_R | aaGGTACCGAGCTTGAGGAACAGACCGCAG |
| ArmDn_Gzf1_NotI_F | aaGCGGCCGCACCGGTGGAGCCAGCACAAACA |
| ArmDn_Gzf1_PfoI_R | aaTCCAGGATGAGGGAAAAAAACTGGGAAAA |
| ArmUp_Azf1_EcoRI_F | aaGAATTCCTAAAAGAGACTATGCATCC |
| ArmUp_Azf1_KpnI_R | aaGGTACCCGCGGTAGTTGCCTCTCTGG |
| ArmDn_Azf1_BamHI_F | aaGGATCCCATGATCAAGTACTTTGGAG |
| ArmDn_Azf1_NotI_R | aaGCGGCCGCCCCCGAAAAACTTCCGAACC |
| ArmUp_Dep1_EcoRI_F | aaGAATTCGAAGGATGACGTGGCAGAGG |
| ArmUp_Dep1_KpnI_R | aaGGTACCGATCTCGGCGAGAGACGTCA |
| ArmDn_Dep1_BamHI_F | aaGGATCCTCAAACTGGCCAAGACGCGT |
| ArmDn_Dep1_NotI_R | aaGCGGCCGCCTGTTGCTGTGAGTATGAGA |
| ArmUp_Euf1_EcoRI_F | aaGAATTCCAGACCCAGCCCCAGTCACA |
| ArmUp_Euf1_KpnI_R | aaGGTACCCGTGCAAACTCTTGGAAGTT |
| ArmDn_Euf1_BamHI_F | aaGGATCCCGTGTCTCTGAACAACTCTA |
| ArmDn_Euf1_NotI_R | aaGCGGCCGCGGAGCGTTAGTCGATTCGTT |
| ArmUp_Cat8_EcoRI_F | aaGAATTCGGTCCAAGAAAATCAAATGCGAC |
| ArmUp_Cat8_KpnI_R | aaGGTACCGTTGACATGCAGCGAATAGGTC |
| ArmDn_Cat8_BamHI_F | aaGGATCCCGGAAGAGAGCAAGAACACACCC |
| ArmDn_Cat8_NotI_R | aaGCGGCCGCCGCATGTTGCAGTGAGCAGCTCC |
| ArmUp_TF024_EcoRI_F | aaGAATTCCCAAAAGTATGTTCTGAGATTTGG |
| ArmUp_TF024_KpnI_R | aaGGTACCGTCACTGGCAGCGGTATCTCCCCC |
| ArmDn_TF024_BamHI_F | aaGGATCCGCCAAAATTGCAGATCTGTCGCGCC |
| ArmDn_TF024_NotI_R | aaGCGGCCGCCGCGAAGCTAAGGAGATGCGATG |
| ArmUp_Brg1_EcoRI_F | aaGAATTCACAAGCAGTTGAACAAGGACATTGG |
| ArmUp_Brg1_KpnI_R | aaGAATTCACAAGCAGTTGAACAAGGACATTGG |
| ArmDn_Brg1_BamHI_F | aaGGATCCCAGATGATCTCGCAACTGGAGACC |
| ArmDn_Brg1_NotI_R | aaGCGGCCGCCTCATTTCGCTCAGAAACCGGTCTC |

**Supplementary Figures**

**Figure S1.** Schematic representation of introns in the mRNAs encoding TFs Klf1, Hac1, and Sfp1, and the activation site introduced to the Hsf1 (R68S) gene variant. The introns were removed, and the 3 bp change was made before cloning the genes for overexpression. Introns are marked in light green, dark green denotes exons, genes’ TSS on the right, the lengths of each part and the whole gene are scaled to actual bp numbers.

**Figure S2.** Schematic representation of a disruption cassette used for the TF-encoding genes deletion. The integration arms spanned the ORF and the regulatory elements regions.

**Figure S3.** Levels of scYFP produced by Y. lipolytica strains modified by OE (closed bars) and KO (open bars) of TFs. The modified and the control strains were cultured under two thermal conditions - 22°C (A) and 28°C (B). Bars show the median of normalized specific fluorescence (nsFL) determined in the biomass (inYFP) or in the culture supernatant at 48 h of culturing. Error bars show the interquartile range (IQR) based on at least 4 replicates. Asterisks show statistical significance (padj < 0.05) of the nsFL values difference between the OE and KO strains, modified in the same TF. The heatmap next to the graph shows relative change (RC) of a given metrics (nsFL for in/outYFP) in the given strain (signatures on the Y axis of the graphs) over the positive control strain (grey closed bar). The heatmap is color-coded according to the legend provided. Whenever the RC value was statistically significant, the value is indicated in the heatmap cell.

**Figure S4.** Levels of scYFP produced by Y. lipolytica strains modified by KO of TFs. The modified and the control strains were cultured under two thermal conditions - 22°C (A) and 28°C (B). Bars show the median of normalized specific fluorescence (nsFL) determined in the biomass (inYFP) or in the culture supernatant at 48 h of culturing. Error bars show the interquartile range (IQR) based on at least 4 replicates. The heatmap next to the graph shows relative change (RC) of a given metrics (nsFL for in/outYFP) in the given strain (signatures on the Y axis of the graphs) over the positive control strain (grey closed bar). The heatmap is color-coded according to the legend provided. Whenever the RC value was statistically significant, the value is indicated in the heatmap cell.

**Figure S5** Levels of scTlG produced by Y. lipolytica strains modified by OE of TFs. The modified and the control strains were cultured under two thermal conditions - 22°C (A) and 28°C (B). Bars show median of normalized specific activity (nsGAU) determined in the culture supernatant at 48 h of culturing. Error bars show the interquartile range (IQR) based on at least 4 replicates. The heatmap next to the graph shows relative change (RC) of a given metrics (nGAU for outTlG) in the given strain (signatures on the Y axis of the graphs) over the positive control strain (grey closed bar). The heatmap is color-coded according to the legend provided. Whenever the RC value was statistically significant, the value is indicated in the heatmap cell.

**Figure S6.** Levels of scYFP produced by Y. lipolytica strains modified by OE (closed bars), KO (open bars), or combination of two TFs OE (closed bars), or an OE and KO of TFs (striped bars). Type of the modification is indicated on the y axis of the graphs. The modified and the control strains were cultured under two thermal conditions - 22°C (A) and 28°C (B). Bars show the median of normalized specific fluorescence (nsFL) determined in the biomass (inYFP) or in the culture supernatant at 48 h of culturing. Error bars show the interquartile range (IQR) based on at least 4 replicates. Grey lines show statistical significance (padj < 0.05) of the nsFL values difference between the OE strain (single modification) or the KO strain (single modification) and the double modification strains (OE-OE or OE-KO), modified in the same TFs. The heatmap next to the graph shows the relative change (RC) of a given metric (nsFL for in/outYFP) in the given strain (signatures on the Y axis of the graphs) over the positive control strain (grey closed bar). The heatmap is color-coded according to the legend provided. Whenever the RC value was statistically significant, the value is indicated in the heatmap cell.

**Figure S7.** Levels of scTlG produced by Y. lipolytica strains modified by OE (single modification) or combination of two TFs OE. Type of the modification is indicated on the y axis of the graphs. The modified and the control strains were cultured under two thermal conditions - 22°C (A) and 28°C (B). Bars show median of normalized specific activity (nsGAU) determined in the culture supernatant at 48 h of culturing. Error bars show the interquartile range (IQR) based on at least 4 replicates. Asterisks show statistical significance (padj < 0.05) of the nsGAU values difference between the OE strain (single modification) and the double modification strains (OE-OE), modified in the same TFs. The heatmap next to the graph shows the relative change (RC) of a given metric (nsGAU for outTlG) in the given strain (signatures on the Y axis of the graphs) relative to the positive control strain (grey closed bar). The heatmap is color-coded according to the legend provided. Whenever the RC value was statistically significant, the value is indicated in the heatmap cell.

**Figure S8**

Specific fluorescence (sFL; A) and fold change of sFL (B) by Azf1- and Dep1-engineered strains and the control strain in continuous bioreactor cultures**.** Statistical analysis was performed in R using the lme4 package. A linear mixed-effects model was employed to account for the nested structure of the data (random effects of biological and technical replicates, strain as a fixed effect). Post-hoc pairwise comparisons were performed using the emmeans package with Tukey’s HSD adjustment for multiple comparisons. Fold changes and their associated 95% confidence intervals were calculated by back-transforming the differences of marginal means from the log 2 scale. Letters a, b, and c indicate homogeneous groups. (C) RNA-seq-derived logFC expression values of the targeted TF genes in the tested strains. Values are shown for Azf1 in Azf-OE and Azf-KO strains, and for Dep1 in Dep1-OE and Dep1-KO strains. The non-zero values of AZF1 and DEP1 expression in the KO strains are non-significant.

**Figure S9**

Morphology of selected *Y. lipolytica* strains – Azf1-OE, Dep1-OE, and the control strain (Y2900).

Details on the strains’ properties were presented (Gorczyca et al., 2024). Biomass of the yeast strains was washed in saline and plated on a microscopic slide. Then fixed. The cells were stained with crystal violet. The images were taken using a ZEISS PrimoStar light microscope under 1000x magnification.

| Azf1-OE | Dep1-OE | Control strain Y2900 |
| --- | --- | --- |
|  |  |  |
